# Bioelectrochemical Systems: Prioritizing Energy Density, Long-Term Stability, and Validation

**DOI:** 10.1021/acsenergylett.5c01678

**Published:** 2025-08-20

**Authors:** Luana C. I. Faria, Steffane Q. Nascimento, Filipe C. D A. Lima, Graziela C. Sedenho, Thiago Bertaglia, Rodrigo M. Iost, João C. P. de Souza, Senentxu Lanceros-Méndez, Shelley D. Minteer, Serge Cosnier, Ariel L. Furst, Frank N. Crespilho

**Affiliations:** † São Carlos Institute of Chemistry, University of São Paulo, 13560-590 São Carlos, SP, Brazil; ‡ Federal Institute of Education, Science and Technology of São Paulo, 15991-502 Matão, SP, Brazil; § Department of Fundamental Chemistry, Institute of Chemistry, University of Sao Paulo, 05508-000 Butantã, SP, Brazil; ∥ Faculty of Sciences, São Paulo State University, 17033-360 Bauru, SP, Brazil; ⊥ Physics Centre of Minho and Porto, Universities (CF-UM-UP) and Laboratory of Physics for Materials and Emergent Technologies, LapMET, University of Minho, 4710-057 Braga, Portugal; # BCMaterials, Basque Center for Materials, Applications and Nanostructures, 48940 Leioa, Spain; 7 Ikerbasque, Basque Foundation for Science, 48009 Bilbao, Spain; 8 Department of Chemistry, Missouri University of Science and Technology, 65409-6518 Rolla, Missouri, United States; 9 Kummer Institute Center for Resource Sustainability, Missouri University of Science and Technology, 65409-6518 Rolla, Missouri, United States; 10 Center for Organic and Nanohybrid Electronics, Silesian University of Technology, Konarskiego 22B, 44-100 Gliwice, Poland; 11 Department de Chimié Moleculaire, CNRS UMR-5250, Université Grenoble Alpes, F-38000 Grenoble, France; 12 Department of Chemical Engineering, Massachusetts Institute of Technology, 02139 Cambridge, Massachusetts, United States

## Abstract

Pioneering work in bioelectrochemistry, particularly the employing of yeast cells to generate electrical current, had substantially favored the comprehension of bioelectrochemical reactions. This foundational research has boosted the development of bioelectrochemical systems (BES), which are significant for sustainable energy solutions. BES technologies, such as biobatteries, biosupercapacitors, and enzymatic and microbial biofuel cells, harness organic and biological systems to provide environmentally-friendly alternatives for energy storage and conversion. Despite their potential, these technologies face challenges in achieving competitive energy densities and long-term stability compared to traditional accumulators and converters. Here, we introduce a new Ragone plot for BES, highlight the pathways to overcome key challenges, and compare BES with traditional technologies. A roadmap outlining future directions for BES development is also presented.

## Graphical abstract

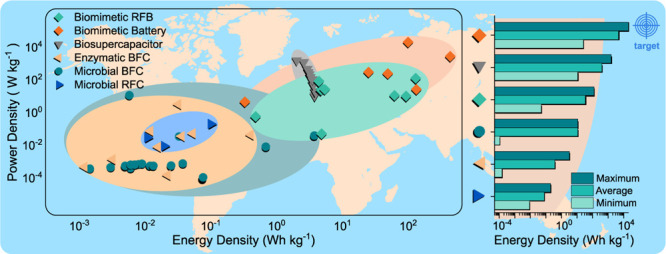

Luigi Galvani’s pioneering work with dissected frog legs marked the origin of modern bioelectrochemistry.^1^ Through a series of experiments, which involved stimulating the muscles of dissected frog legs with electrical currents, Galvani provided evidence of electrical phenomena in biological systems and their role in physiological processes. Building on these initial observations, subsequent researchers began to explore cellular energetics, exemplified by Potter’s experiments with yeast cells.^2^ Potter made significant contributions by investigating the energetics of microbial metabolism using yeast cells as a model system. By constructing a simple setup, Potter demonstrated that yeast cells could generate electrical currents when metabolizing sugars, such as glucose, in the presence of oxygen – an observation that laid the groundwork for what would later become the field of bioelectrochemical systems (BES).

Potter’s experiments were pivotal in highlighting the capacity of biological systems to convert chemical energy into electrical energy through metabolic processes. This realization catalyzed the emergence of BES as a platform for harness biological activity in practical applications, including bioenergy production, biosensing, and medical devices. Figure 1 provides an overview of the main achievements in the area since Galvani’s findings to the advancements in BES technologies in the present day.

**Figure 1:**
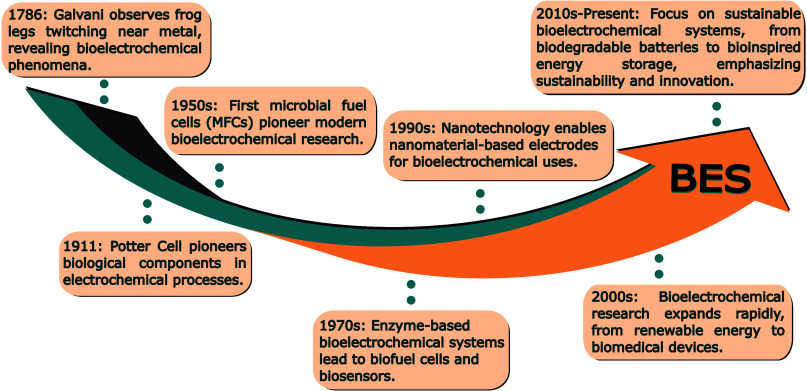
Timeline depicting the progress and achievements in BES from Luigi Galvani’s seminal work to the present day.

More than 100 years after Galvani and Potter’s discoveries, their foundational insights continue to influence the development of both classical electrochemistry and modern BES.^3,4^ Today, sustainability plays an essential role in incentivizing innovation in energy conversion and storage technologies.

The increasing urgent need for clean, renewable, and decentralized energy solutions has led to a growing number of publications and research initiatives focused on BES worldwide. This global effort reflects not only the technological promise of BES, spanning from academia to industry, but also the interdisciplinary nature of the field, which integrates biology, chemistry, physics, engineering, and environmental science. As energy demands rise and environmental concerns intensify, BES are increasingly seen as viable approach to address these challenges.

With worldwide collaborations accelerating innovation and facilitating the creation of more effective, reliable, and sustainable systems, the field has developed into a thriving research environment.^5,6^

## BES Worldwide Landscape

The exponential growth of wearable, flexible, implantable and small electronic devices has created an urgent demand for energy storage technologies that are not only miniaturized but also capable of delivering high performance, long-term durability, and seamless integration. Among the emerging candidates, biobatteries^7–10^ and other bioinspired power sources have attracted considerable attention due their potential to meet these requirements while influencing various sectors, including medical wearables, portable devices, flexible displays, and the expanding Internet of things (IoT) ecosystem. From smart medical patches that continuously monitor physiological signals to ultrathin devices enhancing mobile connectivity, the integration of microscale power sources is a significant enabler of future technologies.^11,12^ Their miniature scale enables seamless integration into device architectures without compromising design flexibility or aesthetic considerations.

The scientific and technological production in different domains of BES technologies (biomimetic redox flow batteries (RFB), biomimetic batteries, biosupercapacitors, enzymatic biofuel cells (BFC), microbial BFC, and microbial redox flow cells (RFC)) has produced a significant number of patents (1,571) and publications (990) in the last five years (Figure 2; see Supporting Information for details on search databases and keywords). These data reflect a growing interest and investment in research into alternative technologies for energy generation and storage, highlighting an emerging trend in exploring innovative methods based on biomimetics, bioelectronics, and enzymatic processes.

**Figure 2:**
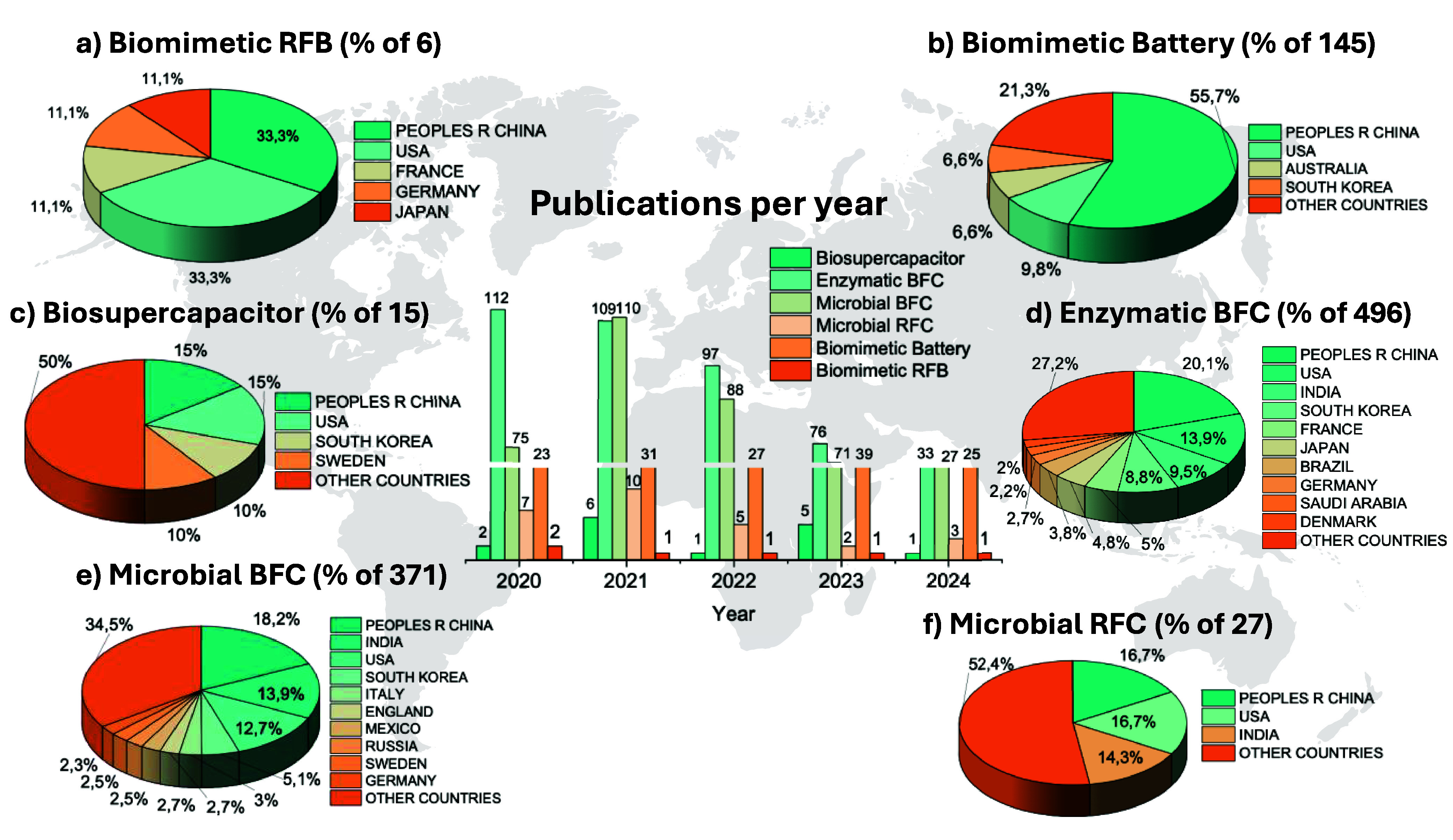
Analysis of the distribution of research, technology, and innovation in BES and Global correlation of publications and deposited patents per country related to (a) Biomimetic RFB, (b) biomimetic battery, (c) biosupercapacitor, (d) enzymatic BFC, (e) microbial BFC, and (f) microbial RFC.

When analyzing the various BES subfields individually, we observe the emergence of pioneering lines of research, such as biomimetic RFB (Figure 2a), biomimetic batteries (Figure 2b), and biosupercapacitors (Figure 2c). These technologies draw inspiration from biological redox processes and natural energy transduction pathways, often leveraging organic molecules or hybrid materials designed to mimic or integrate with biological systems.^13^ While the overall number of publications in these domains remains modest compared to more established fields, the presence of research groups dedicated exclusively to their advancement points to a growing strategic interest and potential for accelerated development shortly. In particular, the use of biomimetic or hybrid catalytic systems in association with enzymes offers promising strategies for efficient energy conversion in reactions involving O_2_ and H_2_.^14–16^ These approaches may lead to the development of cleaner and more sustainable technologies for on-demand power generation in decentralized and miniaturized settings.

In addition to the emerging technologies discussed previously, Figure 2 also presents data on more established BES technologies, such as enzymatic BFC (Figure 2d), which have garnered considerable attention from both academic and industrial sectors, with 496 publications and 598 patents recorded in the past five years. This domain underscores the substantial and ongoing interest in enzymatic pathways for generating bioelectricity. Similarly, microbial BFC (Figure 2e) has attracted significant attention, with 371 publications and 383 patents, reflecting the growing emphasis on microbial metabolism as a sustainable energy source. Although microbial RFC (Figure 2f) remain relatively less explored in the scientific literature – with only 27 publications – they exhibit vigorous innovation activity, with 527 patents, suggesting increasing industrial interest and commercialization potential in this field and reflecting the increasing use of biological materials in energy applications.

Trends in publication data reveal a steep increase in research output across all BES categories, indicating growing global interest and investment in biological-driven energy technologies. Notably, publication rates for enzymatic BFCs have remained relatively stable over the years, suggesting continued advances in enzyme-based energy conversion processes. At the same time, interest in biobatteries (biomimetic batteries and biomimetic RFB) has expanded, supported by growing research aimed at developing sustainable, efficient, and miniaturized energy storage platforms.

Patent data offers additional insights into innovation dynamics and key players within the BES landscape. China and the United States have emerged as dominant leaders in several bioenergy segments, followed by India, South Korea, and Germany, reflecting their significant investments in sustainable energy technologies. Further analysis of the publication types in the area (Figures S1–S6) illustrates the diversity of academic contributions in the field. Articles represent the majority of contributions (990 records), followed by book chapters and conference abstracts. This distribution reflects the interdisciplinary nature of BES research, which encompasses areas such as microbiology applied to biotechnology, biochemistry, molecular biology, enzymology, and electrochemistry, among other disciplines.

Overall, technological analysis suggests that BES technologies are in evidence, and the biological approach is becoming increasingly attractive for clean and sustainable energy production and storage. In the following sections, we summarize the core characteristics and working principles of the main BES technologies under investigation.

## BES Architectures and Operating Principles

As mentioned earlier, BES encompass a diverse set of technologies that integrate biological or bioinspired components into electrochemical energy conversion and storage devices. These systems are often categorized by the type of biological or bioinspired element used (e.g., enzymes, microorganisms, organic molecules) and by their structural architecture.

Here, we detail the operational principles, construction features, and specific configurations of key BES classes: biomimetic RFBs, biomimetic batteries, biosupercapacitors, enzymatic BFCs, microbial BFCs, and microbial RFCs (Figure 3).

**Figure 3:**
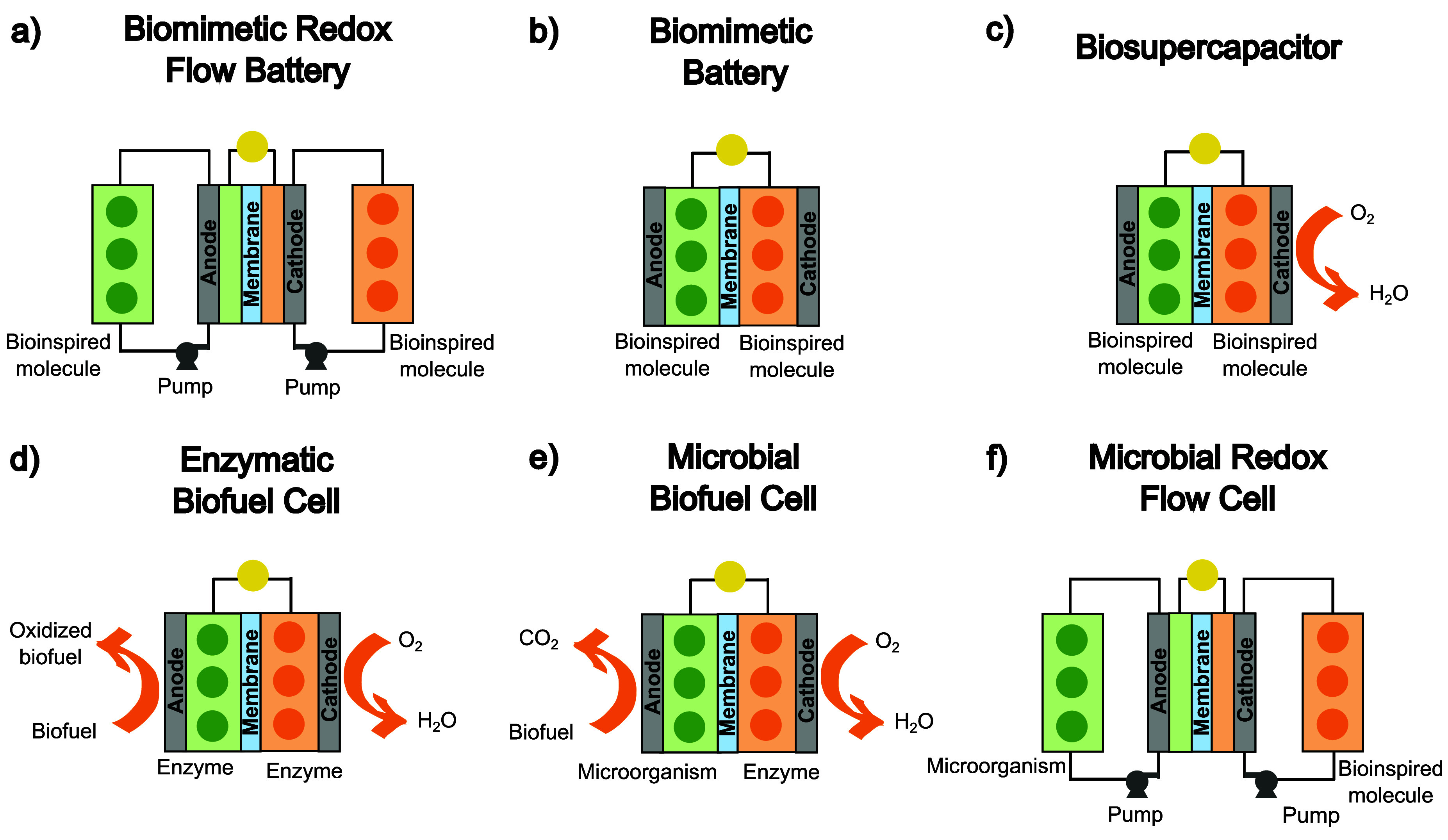
Visual summary of BES architectures and operating principles. (a) Biomimetic RFB, (b) biomimetic battery, (c) biosupercapacitor, (d) enzymatic BFC, (e) microbial BFC, and (f) microbial RFC.

Biomimetic RFBs are rechargeable systems that employ bioinspired redox-active molecules dissolved in liquid electrolytes stored in two separate external reservoirs (Figure 3a). These electrolytes are pumped continuously through an electrochemical cell, where redox reactions occur on the electrodes, discharging chemical energy into an electrical output and reversing the reaction during charging.^17–19^ To preserve electrolyte balance and electric neutrality, a selective ion exchange membrane separates the anode and cathode compartments and selectively permits the cross passage of inactive species.^18^ Unlike traditional batteries, where energy and power are fixed by the cell chemistry, in biomimetic RFBs, the energy capacity is determined by the volume of the electrolyte reservoirs and the power output is governed by the size of the electrochemical cell.^17^

Biomimetic batteries have emerged as a safer and more sustainable alternative to conventional lithium-ion batteries (LIBs), which can pose flammability and environmental risks.^13^ These systems integrate biodegradable, low-toxicity, and renewable materials inspired by biological molecules.^13,20,21^ Typically, they consist of a sealed electrochemical cell with a membrane separating the anode and cathode, both containing bioinspired active materials (Figure 3b). Their design prioritizes eco-friendliness, stationary deployment, and compatibility with a circular economy;^22^ however, limitations such as slower charge rates remain a challenge, which is under active investigation.

Biobatteries and biosupercapacitors differ primarily in their energy and power characteristics.^23^ Biobatteries exhibit relatively high energy densities and can deliver power over extended periods but generally suffer from slow charge rates.^23,24^ In contrast, biosupercapacitors store less energy but excel at delivering high peak power, offering rapid charging capabilities.^23,24^ These devices apply bioinspired or bioactivated materials to store electrical energy through double-layer capacitance and pseudocapacitance mechanisms.^25–27^ These systems usually employ biomolecules, redox enzymes or bacterial cells, etc., immobilized on the electrodes (Figure 3c) to enhance storage and charge transfer, but do not require a continuous fuel supply.^25,28,29^

BFCs are divided into two types depending on the biocatalyst: enzymatic BFCs, which uses redox enzymes to catalyze the electrochemical processes, and microbial BFC, which uses living cells, such as bacteria, fungi and algae.^30^ These BFC categories have been produced through different strategies. While enzymatic BFCs are usually thought of as micropower or potentially nanopower sources, microbial BFCs are typically built as large-scale bioreactors to produce considerable quantities of electrical power.^30^

Enzymatic BFCs convert chemical energy from renewable fuels – such as ethanol, hydrogen, glucose, or xylose – into electrical energy using immobilized redox enzymes (oxidoreductases) as biocatalysts (Figure 3d).^31^ These enzymes are fixed onto the electrode surfaces, enabling direct or mediated electron transfer processes.^31,32^ Operating under physiological pH, ambient pressure, and room temperature, enzymatic BFCs are particularly well-suited for biomedical applications, including powering implantable devices and biosensors.^31–33^ However, enzyme instability and limited operational lifespan remain significant barriers to commercial viability.

Microbial BFCs utilize anaerobic reactions catalyzed by microorganisms (e.g., bacteria, fungi, algae) to drive the oxidation of organic substrates such as lactate, acetate, or glucose.^34^ These systems typically consist of two compartments (anode and cathode) separated by a membrane. At the anode, electroactive microorganisms oxidize organic matter, releasing electrons that travel through an external circuit to the cathode (Figure 3e).^35^ Simultaneously, protons generated at the anode pass through the membrane to react with oxygen and electrons at the cathode, forming water.^34^ Microbial BFCs are often designed as large-scale bioreactors, offering a promising route for simultaneous wastewater treatment and power generation.^36^

Microbial RFCs combine the scalable architecture of biomimetic RFB with the biological catalytic power of microorganisms. In this system, microbial RFC produces electrons by converting organic biodegradable chemicals into electroactive microorganisms (Figure 3f).^37,38^ The electricity is produced when these electrons move from the anode to the cathode. As with biomimetic RFBs, a membrane separates the anode and cathode compartments, and system energy and power can be independently tuned – the former by adjusting the volume of stored electrolyte and the latter via the size of the cell stack.^37^ Microbial RFCs uniquely integrate the biological regeneration of redox-active fuels, creating a self-sustaining and renewable platform for energy production.

Considering these bioinspired systems, rational design strategies for biomolecules, such as enzymes and redox proteins, have been used to increase their efficiency, selectivity, and operational stability. Molecular engineering procedures, such as modifying enzymes to facilitate direct electron transfer can improve system efficiency. In biomimetic RFBs and biomimetic batteries, the rational design of biomolecules, such as quinones, flavins, and phenazines, has enabled improvements in solubility, reversibility, and molecular size.^39–42^ In biosupercapacitors, enzymes and redox proteins can be more frequently used due to the fact that they enable the coupling of energy conversion and storage from biocatalytic reactions.^43,44^ In enzymatic BFCs, glucose oxidase (GOx) and bilirubin oxidase (BOD) are frequently used enzymes. Rational modifications of BOD have been reported to improve direct electron transfer, as well as improve pH and temperature fluctuations.^45,46^ In microbial BFCs, electroactive bacteria, such as *Geobacter sulfurreducens* and *Shewanella oneidensis*, can use redox proteins, such as cytochrome c, to improve electron transfer.^47^ Finally, in microbial RFCs, biomolecules must be compatible with the metabolic activities and electron transfer of microorganisms, as well as redox mediators that facilitate this transfer, such as cytochromes, flavins, quinones and extracellular polymeric substances (EPS).^37,38,48^

Thus, the importance of the interaction between biomolecules and electrode surfaces is evident, especially for direct electron transfer. Carbon-based materials, such as felt, paper and fiber, have been extensively used in biobatteries due to their high surface area and good conductivity.^49–52^ Biosupercapacitors seek materials with high electrical conductivity and large surface area, examples of which include activated carbon, graphene, carbon nanotubes and carbon fabrics.^53–56^ For use in enzymatic BFCs, electrodes are selected based on their compatibility with enzymes, chemical stability, and conductivity, examples of suitable electrodes include carbon nanotubes, electrodes functionalized with redox polymers, and electrodes modified with metal nanoparticles.^57–59^ In microbial BFCs, it is important to consider the electrode’s biocompatibility with microorganisms, thus gas diffusion electrodes, carbon nanotubes, activated carbon, and catalyst-coated graphite are commonly used.^60–62^ Finally, for Microbial RFCs systems, the electrodes need to fulfill two functions: biofilm support and compatibility with redox species, such as carbon felt, carbon nanomaterials, activated carbon with binder, electrodes modified with metal oxides, among others.^37,63^ Thus, it is important to recognize that the integration of biomolecule engineering and electrode modeling are fundamental for the advancement of BES technologies, as well as their implementation.

From this perspective, the advancement of BES technologies is intrinsically associated with the development of specific materials that address their weaknesses. An example is the integration of 3D electrodes in BES devices, as the increased surface materials of these structures provide some advantages. In general, 3D electrodes shorten the diffusion path of biomolecules (or fuels) and higher biocatalyst loading, directly impacting the performance of BES devices. For instance, third-generation electrodes were introduced to energy harvesting in photoelectrochemical systems in 2015.^64^ Those electrodes, named IO-mesoITO, are composed of an inverse opal-based indium tin oxide (ITO) supported in a fluorine tin oxide-covered glass. This demonstrated the production of a light-driven BFC by coupling photosystem II (PS II) immobilized into IO-mesoITO and a hydrogenase cathode.^64^ The same authors addressed the improved performance of such electrodes in subsequent studies.^65,66^ In other example, the impact of 3D structures on the performance of a lactate oxidase (LOx)-based wearable BFC was evaluated. By mixing styrene-ethyl butylene styrene (SEBS) with multiwalled carbon nanotubes (MWCNTs) followed by a nonsolvent induced phase separation (NIPS) approach, a porous, flexible 3D interpenetrating network capable of hosting high amounts of Lox was presented. Thanks to the increased surface area, improved mass transport, and higher enzyme loading, the wearable BFC developed delivered an unprecedented power of 1.6 mW cm^–2^ and presented an energy density equal to ∼1.38 mWh, underscoring the beneficial use of 3D electrodes in BFCs.^67^ Biotemplating, which consists of using natural structures to produce functional materials at the nanoscale, is another recent strategy that could improve the overall performance of BES devices. This approach allows us to produce uniform structures with controlled size and, consequently, to design specific materials for the desired application.^68^ This bioinspired approach is becoming increasingly popular in the field of lithium and sodium-ion batteries;^68^ however, it has barely penetrated the field of bioelectrochemistry,^69^ which represents the opportunity for the development of tunned materials for addressing the weaknesses of BES devices.

Mesoporous carbon-based electrodes stand out as interesting materials to increase the power output of BFCs, as highlighted by earlier reports on H_2_/O_2_ BFCs. For instance, Lojou and co-workers developed a mesoporous carbon-based electrode by performing layer-by-layer deposition of carbon nanotubes (CNT) into a carbon felt (CF), followed by modification with amino-pyrene derivatives.^70,71^ This simple yet highly efficient electrode enables the preferential orientation of enzymes on the electrode surface, favoring their direct electrical wiring and, consequently, improving their bioelectrochemical performance.^70,71^ In a following study, the authors expanded this approach by using a CF-CNT electrode modified with aminomethylpyrene (PyrNH_2_) for immobilizing two thermostable enzymes, namely *Aa* MBH (hydrogenase) and *Bp* BOD (multicopper oxidase).^72^ The CF-CNT electrode showed a porous size ranging from 10 to 40 nm, a similar size to the chosen enzymes, suggesting this electrode is a suitable platform for entrapping such enzymes, creating high-performance bioelectrodes. By coupling *Aa* MBH and *Bp* BOD immobilized electrodes and using a convective supply of substrate, the authors obtained an impressive power output of 1.7 mW cm^–2^ at 50 °C (81 mW cm^–3^), an outstanding power output for bioelectrochemical devices. Also, after 17h working at a load of 1.5 mA, this BFC delivered 15.8 mWh with only 5% power loss, demonstrating its remarkable stability. Further measurements in quiescent solution, however, demonstrate a much smaller power output, evidencing mass transport as a limiting factor of such a system.

Similarly, Kano and co-workers demonstrated the development of engineering waterproof carbon cloth electrodes (WPCC) for achieving high-power H_2_/O_2_ BFCs.^73,74^ The first study deals with developing a dual gas-diffusion H_2_/O_2_ BFC by applying a WPCC Ketjen black-modified gas diffusion electrode for immobilizing BOD and an oxygen-resistant hydrogenase (DνMF).^74^ Although the authors did not assemble a full BFC due to the risk of explosion, the electrochemical characterization of both electrodes suggests the proposed dual gas system could deliver a power of 8.4 mW cm^–2^ with a voltage output of 1.14 V. In a later work, the authors demonstrated the feasibility of the dual gas-diffusion device by coupling BOD and hydrogenase electrodes into a single device separated by 1.5 mol L^–1^ citrate solution.^73^ In such a device, air and pure H_2_ were supplied at the cathode and anode, respectively, and the whole device worked at quiescent conditions and room temperature. Notably, engineered WPCC surfaces with negative and positive net charges were employed, aiming to achieve a preferential conformation when immobilizing the enzymes. Again, this device showed unprecedented electrochemical performance, delivering 6.1 mW cm^–2^ at 0.72 V and an open circuit voltage (OCV) of 1.12 V.

Improving BES performance requires an in-depth understanding of the mechanisms underlying the electron transfer (ET) between the biocatalyst and electrode surface. In this regard, kinetic modeling of bioelectrochemical processes is essential. Hydrogenases are hypothesized to play an important role soon due to the increasing worldwide need for green fuels, such as hydrogen. Consequently, understanding the kinetics parameter governing its bioelectrocatalysis could benefit the energy conversion and storage field. Recent works in the literature showed that simple one-electron models are not enough to explain the voltammetric responses of bidirectional catalysts.^75^ Instead, the position of catalytic potentials, both reduction and oxidation, directly depends on the reduction potentials and proton affinities of key intermediates, as well as the rate constants of chemical steps in the catalytic cycle.^76^ It explains why some enzymes show reversible catalysis while others are more irreversible, even if the mechanistic cycle is basically the same. Fasano et al. propose that the separation between the emergency on catalytic oxidation and reduction defines how reversible the catalysis is, which is directly related to energy efficiency.^77^ Additionally, modeling the voltammetry of adsorbed enzymes must also consider heterogeneous electron transfer rates at the interface and possible limitations along intramolecular ET chains.^78^ Overall, these models help to understand how changes in pH, electrode potential or the enzyme environment impact the device performance.

Although a long path to commercial success of BES might occur, the use of functional materials designed on purpose to address the weaknesses of these technologies could shorten it, as the previously highlighted studies have shown. Equally important is the theoretical understanding of the mechanisms underlying the bioelectrochemical processes, such as the catalytic mechanisms of enzymes or protein complexes and ET transfer pathways from electrogenic microorganisms. Indeed, the information obtained by one field will feed insights to another, helping to create a fruitful cycle, which may propel BES devices toward improved performance. This elaborate cycle is important not only to expand our knowledge about BES but also to expand the devices commercially available. Addressing the weaknesses of BES devices is ultimately an energy transition strategy toward a greener future.

## Biobatteries

Biobatteries^7–10,49^ have emerged as promising candidates for sustainable energy and conversion, leveraging organic and organometallic molecules to achieve efficient power generation. Results from various studies highlight the diverse capabilities of biobatteries across different configurations and performance metrics (see Table S1 and S2). Here, we categorize biobatteries into two distinct classes: biomimetic RFBs and biomimetic batteries.

One notable example is a system based on biologically inspired pteridine redox centers for rechargeable batteries, which achieved a specific energy of 348.0 Wh kg^–1^ and a specific power of 20.0 kW kg^–1^.^8^ Similarly, wearable and washable fiber zinc batteries with a specific energy of 264.7 Wh kg^–1^ and a specific power of 32.6 W kg^–1^ are ideal for wearable power textiles.^9^ All-organic aqueous batteries powered by adsorbed quinone promote a specific energy of 25.0 Wh kg^–1^ and a specific power of 290.0 W kg^–1^, demonstrating the feasibility of a bioinspired interface design for an efficient energy storage system.^10^ These findings show the potential of biobatteries in various applications, ranging from microscale devices to wearable electronics,^79^ offering sustainable and environmentally friendly alternatives for energy storage.

Another relevant class within biobatteries are RFBs, which also use bioinspired molecules such as phenazines,^80–82^ flavins,^83^ alloxazines,^84^ and quinones.^49,85–87^ A biomimetic RFB using flavin as an electrolyte achieved a peak power density of 160 mW cm^–2^ at a current density of 300 mA cm^–2^, which is higher than the all-vanadium RFB.^83^ These findings underscore the viability of incorporating bioinspired redox chemistries into scalable energy systems.

## Biosupercapacitors

In terms of biosupercapacitors,^43,88–97^ Tables S3 and S4 summarizes the power density values reported for various device configurations. These configurations encompass self-charging biocapacitors,^88^ supercapacitor/biofuel cell hybrids,^89^ Nernstian biosupercapacitors,^90^ ceramic microbial fuel cells operating in supercapacitive mode,^91^ biosupercapacitors for powering oxygen sensing devices,^92^ and biofuel cell/self-powered hybrid μ-supercapacitors,^93^ among others.^43,94–97^ Power densities ranging from 0.87 mW cm^–2^ to 25.52 mW cm^–2^ have been achieved in these systems, demonstrating the versatility and potential of biodevices for energy conversion and storage applications.^43^ Several factors contribute to the variation in power density, including electrode area, electrolyte volume, temperature, and electrode materials. For instance, an implantable antibiofouling biosupercapacitor achieved a remarkable power density of 25.52 mW cm^–2^.^96^ Similarly, the highly sensitive and stable fructose self-powered biosensor based on a self-charging biosupercapacitor demonstrated a power density of 3.8 mW cm^2^ mM^–1^, highlighting the importance of efficient energy conversion in biosensing applications.^97^

## Biofuel Cells

Enzymatic BFCs^98–107^ have emerged as promising BES devices for various applications, including implantable biomedical devices and portable electronics.^33^ The results presented encompass diverse configurations and operational conditions, showing applications across different scales and environments (see Table S5). In 2010, the first example of a BFC implanted in the abdomen of a rat was described, with the animal awake and free to move while the biofuel cell performance was determined.^108^ The first implanted glucose biofuel cell (GBFC), using bodily fluids from mammals, serves as a power source for electronic devices capable of powering a light-emitting diode (LED) or a digital thermometer. When placed inside a rat’s abdomen, the GBFC generates an average open-circuit voltage of 0.57 V. The power output of this implanted GBFC was 38.7 μW, which translated into a volumetric power of 161 μW mL^–1^ and a power density of 0.1935 mW cm^–2^.^109^ Implantable configurations,^98,99^ such as those designed for rats and insects, demonstrate significant power densities ranging from 0.055 mW cm^–2^ to 0.0950 mW cm^–2^, with corresponding volumetric power outputs of 0.475 W m^–3^ to 7.8 W m^–3^, respectively.^98,99^

Miniaturized designs operating at pH 5 and pH 7 exhibit power densities of 0.137 mW cm^–2^ and 1.25 mW cm^–2^, with volumetric power outputs of 1.37 W m^–3^ and 3.20 kW m^–3^, respectively.^100,104^ Hydrogel-based systems,^101^ such as viologen hydrogel at pH 7, offer competitive power densities of 0.178 ± 19 mW cm^–2^ and volumetric power outputs of 35.6 W m^–3^. Advanced configurations, such as those employing carbon nanotubes (CNTs) and hydrogenase/polymer or glucose oxidase/polymer combinations,^102,103^ demonstrate impressive power densities ranging from 0.530 mW cm^–2^ to 2.18 mW cm^–2^, corresponding to volumetric power outputs of 53 W m^–3^ to 4.3 W m^–3^, respectively. These results underscore the versatility of enzymatic BFC in various applications, ranging from medical implants to portable electronics, offering high power densities and energy outputs in compact and efficient configurations.

Regarding microbial BFC, as presented in Table S6,^36,110–120^ it offers various configurations pertinent to wastewater treatment and biomass applications. Several electrode areas and electrolyte volumes have been explored, resulting in a substantial range of power density outcomes. For instance, configurations utilizing larger electrode areas and electrolyte volumes, such as 1 m^3^, have demonstrated power densities ranging approximately from 0.3 to 33.5 W m^–3^, indicating the potential for scalable energy generation from wastewater processes. Conversely, smaller-scale setups, such as those with a 45.0 L electrolyte volume, have exhibited power densities around 0.5 W m^–3^, indicating efficient energy conversion even at reduced scales.^113^ The influence of electrode materials and temperature control on power density outcomes can also be considered. For example, configurations employing electrode areas ranging from 2482.0 cm^2^ to 600.0 cm^2^ have yielded power densities spanning from 7.3 to 18.1 mW cm^–2^, illustrating the impact of electrode size on energy conversion efficiency.^113,114^ Furthermore, variations in electrolyte volume and operating temperature enable tuning of power density ranges. Additionally, specific configurations have allowed for a power density of 11.22 ± 0.7 kW m^–3^, underscoring the potential of biomass-based energy conversion technologies to deliver high power outputs.^116^

## Microbial Redox Flow Cells

In microbial RFC (see Table S7), the energy conversion process includes processes such as methane production at biocathodes and the involvement of microaerobic iron-oxidizing bacteria. The key results include the operation of BES as bioanodes and biocathodes in the microbial RFC, utilizing redox pairs such as anthraquinone-2,6-disulfonate (2,6-AQDS) and ferricyanide ([Fe­(CN)_6_]^3–^) for energy conversion.^37^ A current density of 0.048 mA cm^–2^ with a bioconversion rate of approximately 27% for the reduction of 2,6-AQDS to 2,6-AQDSH_2_ was achieved. Additionally, 35.7% of [Fe­(CN)_6_]^4–^ was oxidized to [Fe­(CN)_6_]^3–^. The microbial RFC, operating for 29 cycles, achieved Coulombic efficiencies of around 99% and energy efficiencies of approximately 55%. *Geobacter sulfurreducens*, an electroactive bacterium, was also utilized to charge 2,6-AQDS, resulting in current densities of approximately 200.0–500.0 mA m^–2^ and maximum power densities of around 0.0033 mW cm^–2^.^38^ Furthermore, the microbially charged electrochemical fuel, in combination with [Fe­(CN)_6_]^3–^, yielded a potential difference of 0.62 V and achieved an energy conversion efficiency of about 80%. Envisioning the use of a BES for charging the positive electrolyte of an RFC, the study suggests the potential for bioconversion of waste biomass energy into electrochemical fuels for generating electricity.

Beyond power output, methane production at biocathodes represents an approach to storing renewable electrical energy as chemical energy through the biological conversion of carbon dioxide.^121^ Methane-producing microorganisms utilize electricity to catalyze the conversion of carbon dioxide into methane, a form of carbon-neutral natural gas.^122^ The design features a high area-to-volume ratio of 2.0 cm^2^ cm^–3^ and an external capillary manifold for flow distribution, allowing for current densities up to 3.5 mA cm^–2^ and resulting in volumetric methane production rates of up to 12.5 L CH_4_ L^–1^ d^–1^.^122^ The high area-to-volume ratio and efficient flow distribution provided by the RFB design contribute to the improved performance and increased methane production rates in the BES. Microaerobic ferrous-oxidizing bacteria (FeOB) were selectively enriched on the surface of graphite felt using Fe^2+^-diethylenetriaminepentaacetic acid (DTPA) as the energy source. FeOB contributes to improving the performance of all-iron flow batteries in several ways, including promoting the oxidation of Fe^2+^-DTPA, leading to a higher rate of Fe^2+^ oxidation compared to a simple chemical process.^123^ The experimental reactors achieved a maximum current density of 2.256 mA cm^–2^ at 0.1 mol L^–1^ electrolyte concentration. Additionally, the power density of the experimental reactors was reported to be 0.342 mW cm^–2^. The specific capacity of the all-iron flow battery increased with the presence of ferrous-oxidizing bacteria, particularly at a 0.3 mol L^–1^ electrolyte concentration and 10.0 mA cm^–2^ current density.

## Benchmarking the Performance of Bioelectrochemical Systems

Recent studies have demonstrated significant progress in the BES field, with notable advancements in power output, current density, and energy efficiency.^6,124,125^ Different BES configurations revealed power outputs ranging from 10.0 to 1000.0 mW cm^–2^ and current densities between 0.0050 and 0.0500 A cm^–2^. These findings highlight the variability in performance across different systems and underscore the importance of standardized production and testing protocols. In addition to electrical performance metrics, benchmarking efforts often assess the substrate removal rates and treatment efficiencies of BES for wastewater treatment applications.^6,126–128^ Studies have reported chemical oxygen demand removal rates ranging from 24% to 97%.^36,110–114,117^ Furthermore, comparisons with conventional treatment technologies have shown that BES can achieve comparable or even superior removal efficiencies, particularly for recalcitrant compounds and emerging contaminants.

Pilot-scale demonstrations have provided further insights into the scalability and practical feasibility of BES technologies. For example, a pilot study conducted at a wastewater treatment plant demonstrated the successful integration of a BES unit for enhanced organic removal and energy recovery.^126^ The system achieved chemical oxygen demand (COD) removal efficiencies exceeding 80% while generating electricity with an average power output of 5.0 kW. These results highlight the potential of BES for decentralized wastewater treatment and energy recovery applications. Moving forward, continued investment in benchmarking research and collaborative initiatives is essential for accelerating the development and commercialization of BES technologies. Biomimetic RFB, biomimetic batteries, biosupercapacitors, enzymatic BFCs, microbial BFCs, and microbial RFCs represent distinct avenues within BES, each offering advantages and challenges in energy conversion and storage. Each type of BES exhibits distinct capabilities and applications, ranging from high specific energy outputs in biomimetic batteries^129,130^ to scalable energy generation in microbial BFCs^36,110–114,117^ and innovative energy conversion mechanisms in microbial RFCs.^37,38,121,123^ These comparative analyses highlight the diverse potential of BES technologies in addressing energy challenges and advancing sustainable energy solutions (See Figure 4).

We conducted a nonsystematic survey of the literature, selecting representative studies across a broad range of BES without predefined criteria or bias toward specific device types. The goal was to capture a wide spectrum of reported performances and configurations, enabling the construction of a comprehensive comparative analysis for each BES category.

Thus, to construct Figures 4 and 5, our approach aimed to reflect the overall landscape of published research and highlight the diversity of design strategies and performance metrics reported in the field.

**Figure 4:**
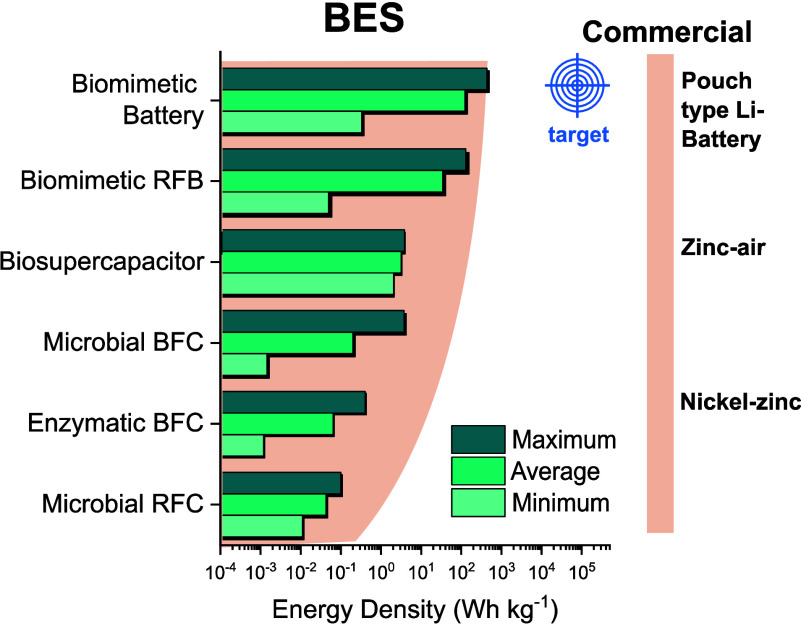
Correlation between energy density and BES types. On the left are the biodevices, and on the right, there is a direct correlation with available market technologies. The values obtained are correlated with the maximum energy density for each device provided by specialized companies and the literature. The biodevices data are based on the Ragone plot obtained in this study, as described in the Supporting Information.

**Figure 5:**
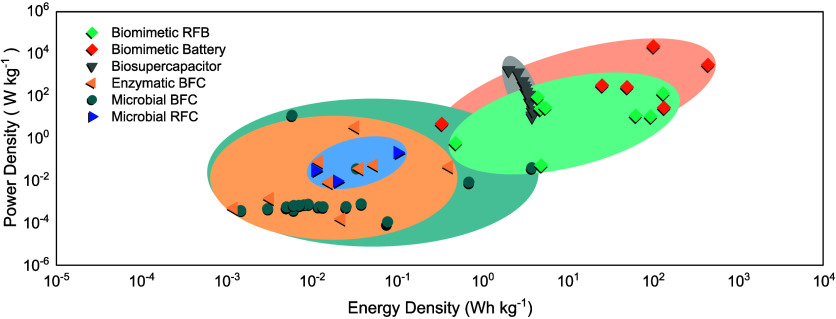
Bio-Ragone plot for the studied BES. The spheroids indicate potential limits based on the results compiled in this work.

Biobatteries harness organic and organometallic molecules to generate power efficiently.^41,130,131^ Gel-based microbatteries exhibit notable capacities and energy outputs, while biologically inspired redox centers and fiber zinc batteries offer specific energy tailored for different applications.^7,132^ However, all-organic aqueous batteries present comparatively lower specific energy due to the potential limitation imposed by the water stability window. Biosupercapacitors demonstrate versatile power density values across various configurations, ranging from self-charging biosupercapacitors to supercapacitor/BFC hybrids.^133,134^ Noteworthy examples include implantable biosupercapacitors with remarkable power densities and self-powered biosensors, which highlight efficient energy conversion in biosensing applications.

Enzymatic BFCs show significant power densities across diverse designs and operational conditions. Miniaturized configurations and advanced setups utilizing carbon nanotubes exhibit high power densities and volumetric power outputs, emphasizing their versatility in biomedical implants and portable electronics.^70,71,135–137^ A frequently raised concern regarding the practical application of enzymatic BFC is the cost of redox enzymes, particularly when highly purified or recombinant forms are used. However, achieving high specific current densities and long-term stable electrodes can significantly and positively impact the cost-performance balance of enzymatic BFCs.^70,71,137–141^ For example, engineering electrode interface with nanomaterials and functionalization yielded milliampere-order current magnitudes using nanomol of enzymes.^70,71,137–139^

Microbial BFCs offer scalable energy generation potential from wastewater processes, with power densities influenced by electrode area and electrolyte volume.^142^ Ceramic microbial fuel cells and biomass applications underscore the importance of material selection and design optimization in augmenting energy conversion efficiency.^143,144^ Microbial RFCs have been used for energy conversion and storage, with methane production at biocathodes and microaerobic ferrous-oxidizing bacteria enabling enhanced performance.^121,122^

One of the main challenges for BES is achieving competitive energy density and power density levels comparable to those of conventional batteries. While BES offer sustainability benefits, their energy outputs may not yet meet the demands of high-power applications, such as electric vehicles or grid-scale energy storage. Additionally, comparisons between BES technologies and conventional sustainable energy technologies often underestimate the potential utility of biological systems because of the well-established benchmarks and metrics for abiotic technologies. Systems ranging from batteries to photovoltaics have defined characterization data required for reporting or publication of new platforms. This consensus within the field has supported innovation by making improvements well-defined. For BES technologies to achieve similar status among clean energy technologies, equivalent benchmarks are needed for direct comparisons.

## Targets for Bioelectrochemical Systems

Numerical targets serve as benchmarks for researchers, engineers, and policymakers, providing clear goals to aim for across various types of BES. In this way, we constructed a bio-Ragone plot for BES systems (Figure 5), plotting power density against energy density, which enables the comparison of different device performance.

For biomimetic batteries, specific energy should achieve at least 300.0 Wh kg^–1^ to match conventional lithium-ion batteries.^145^ The power density is targeted at 1.50 kW kg^–1^ for rapid energy delivery in high-power applications.^146,147^ Energy efficiency aims for 90% or higher to maximize chemical energy conversion into electrical energy. Biosupercapacitor power density should attain a considerable power density to support rapid charge and discharge cycles. Capacitance targets 50.0 F g^–1^ to maximize energy storage capacity while maintaining compact device dimensions.^43^ Self-charging capability aims to develop biosupercapacitors capable of self-charging through ambient environmental conditions, such as light or temperature gradients. For Enzymatic BFCs, power density targets 1.3 mW cm^–2^ to enable efficient energy generation for portable electronics and biomedical implants.^104^ Longevity aims for a lifespan of at least 12 months^148^ under continuous operation. Fuel flexibility targets BFCs capable of utilizing a wide range of biocompatible fuels, including glucose, lactate, and hydrogen, for versatility and adaptability. In the domain of microbial BFCs, power output reaches 50.0 W m^–3^ in systems operating with wastewater^112^ and 11.22 kW m^–3^ in miniaturized systems,^116^ both of which serve as a fuel source to enable scalable energy generation for decentralized applications. COD removal efficiency targets 95% or higher to ensure effective wastewater treatment while generating electricity. Stability aims to maintain a stable power output over 12 months under varying environmental conditions demonstrating long-term reliability and robustness. For microbial RFCs, the methane production rate remains modest, around 60.0 L CH_4_ m^–2^ day^–1^, utilizing anaerobic microorganisms for efficient energy conversion.^122,149^ Coulombic Efficiency aims for 80% or higher to maximize electron recovery from microbial metabolism and minimize energy losses. Scalability targets the development of scalable microbial RFC systems capable of operating in both laboratory-scale reactors and field-scale applications for practical implementation.

Optimizing the performance of BES for improved long-term stability and commercial viability involves several key strategies. Reducing leakage current, charge redistribution and side Faradaic reactions is essential. Addressing these factors will enhance the overall performance and feasibility of BES as a sustainable energy solution. The above indicated numerical targets provide a roadmap for researchers and stakeholders to prioritize research efforts, optimize system designs, and assess the performance of BES in achieving sustainable energy solutions.

## Prioritizing Validation and Standardization

The primary conclusion drawn from the data is that while various types of BES, including biomimetic RFB, biomimetic batteries, biosupercapacitors, microbial BFCs, enzymatic BFCs, and microbial RFC show potential for long-term operation and commercial viability, each type faces distinct challenges primarily related to the chemical stability of active materials, standardization of reporting, and economic feasibility. Enhancing the stability and performance of these systems requires comprehensive molecular engineering and interdisciplinary collaboration to develop new materials and optimize existing ones. Additionally, addressing high production costs, particularly for membranes and enzymes, and improving operational stability through advanced fabrication techniques and material innovations are crucial steps toward their commercial application.

The long-term stability of biomimetic RFB systems is primarily influenced by the chemical stability of the active reagents, as these molecules experience capacity loss due to side reactions in the electrolyte. Researchers are extensively studying the molecular decomposition mechanisms of these compounds to better understand the loss or reduction in redox activity,^41,150,151^ aiming to enhance the long-term stability of these systems. RFBs generally have a lifespan of 10 to 15 years, depending on the redox species involved. This estimate is based on the capacity loss rate of organic molecules, which can be categorized as ″high″ (>1.0% day^-1^), ″moderate″ (0.1–1.0% day^-1^), ″low″ (0.02–0.1%), and ″extremely low″ (≤0.02%).^150^

There are still gaps in academia regarding the standardization of RFB data reporting.^152^ Current studies suggest that presenting capacity loss rates over time rather than by cycle is more suitable, as it directly correlates with the decomposition of the active redox species.^150,151^ Additionally, the method of cycling, potentiostatic with current cut-offs versus galvanostatic with potential cut-offs, affects the observed capacity, with the former allowing closer to 100% theoretical capacity access.^151^ The primary limiting factor for aqueous biomimetic RFB commercialization is the thermodynamic potential window of water, approximately 1.23 V, as reactions exceeding this window can compromise the cell’s Coulombic efficiency and operational lifespan.^81,153^ Researchers are working on strategies to bypass the water potential window, aiming to maintain slow water kinetics, which would allow for higher voltage and greater energy and power density, ultimately reducing costs. Despite their advantages, such as low cost, scalability, biodegradability, and environmental benefits, organic molecules possess highly adaptable chemical and physical properties. Thus, the performance of biomimetic RFBs can be optimized through molecular engineering, which facilitates the development of new organic molecules and enables the adaptation of solubility, redox potential, and molecular size.

The long-term stability of biomimetic batteries is limited by the chemical stability of the molecules used. Quinones, the primary class of organic molecules employed, suffer from capacity loss due to side reactions in the electrolyte, such as the formation of anthrones from dimerization, which leads to a loss of electroactivity. These molecules are often used at extreme pH levels to achieve higher capacities. However, these conditions can induce hydrolysis or new group insertions, altering the electrochemical potential and performance of the battery. Some studies report lifespans of up to 600 cycles, while longer lifespans of 4,000 ^154^ and 5,000 ^155^ cycles have also been reported. These values are comparable to those obtained by emerging technologies, such as sodium and zinc-ion batteries, indicating that biomimetic batteries are commercially promising. Most studies normalize capacity and power values based on the mass of active compounds, which is not aligned with industry protocols that use the total device weight for normalization. Standardizing data reporting by the total device weight would enable direct and clear comparisons between current and emerging energy storage technologies, potentially enhancing industry-academia integration and accelerating the adoption of new materials on a large scale.

The low capacities obtained are a primary limiting factor for the commercial application of biomimetic batteries. The use of ion-exchange membranes instead of polymeric separators, as in intercalation batteries, also imposes additional costs. The chemical and commercial viability of biomimetic batteries depends on developing new materials. This requires cooperation among professionals from various fields, such as chemists, physicists, biologists, materials scientists, and engineers. Collaboration between basic and applied science professionals will lead to a deeper understanding of development and application issues. Potential strategies include developing highly soluble and stable organic molecules in aqueous environments, utilizing high-surface-area current collectors/electrodes, and employing hydrogels or 3D electrodes to enhance power density. The involvement of environmental scientists and biologists is crucial for developing devices that minimize environmental damage and for leveraging their knowledge of biological systems in designing innovative electrochemical materials.

Factors affecting the long-term stability of BES systems are not reported in the literature for biosupercapacitors. Biosupercapacitors have operational lifespans reported in days, with some devices retaining 49% of their initial capacitance after 8 days.^43^ Others maintain operational stability for 2 to 3 days.^90,156^ For biosupercapacitors, weighing the device to determine the real energy and power density is needed, given the varying materials and configurations used in these devices. Durability and the high cost of using purified enzymes are major obstacles to commercialization. To commercialize BES, it is necessary to reduce leakage current, charge redistribution, and faradaic side reactions while increasing the lifespan.

The operational stability of enzymatic BFCs can last up to one year.^148^ High operational stability loss and power output decline within the first days of operation are common,^157^ influenced by enzyme loading, temperature, substrate concentration, and pH.^158^ While operational stability can last up to one year, no longer studies have been reported.^148^ Reported parameters include current, current density, open circuit potential (OCP), OCV, mass per electrode area, and power density.^157,159,160^ Improved comparison requires consistent reporting of active surface area, enzyme activity, temperature control, and detailed experimental conditions.^161^ Challenges include enzyme system stability, operational storage, reproducibility across different geographic areas, and disposal issues involving micro- and nanomaterials.^161^ Stability can be improved through suitable biomolecule immobilization,^162^ membrane development, biocatalysis regeneration, miniaturized system design,^163^ bioengineering of enzymes and proteins,^164^ and extensive testing under real operating conditions for large-scale production.^104^

The performance of enzymatic BFC is influenced by several factors, with the most crucial being the intrinsic enzyme properties and enzyme immobilization on the electrode surface. The catalytic efficiency, substrate specificity, operational stability, and electron transfer mechanism of the enzyme are central to determining the overall performance of an enzymatic BFC. Enzymes such as GOx, alcohol dehydrogenase (ADH), laccase, and BOD are commonly used in anodes and cathodes of enzymatic BFC due to their well-characterized redox properties. The immobilization of these redox enzymes on the electrodes should favor the direct electron transfer from their active sites, providing faster and more efficient charge transfer without the need for external mediators. This approach significantly enhances current density and energy output, simplifies cell design, and improves biocompatibility.^165^ In addition, in the cases of NAD-dependent enzymes, such as ADH, the functional groups on carbon electrodes should promote effective coenzyme regeneration.^166^

An alternative approach is to combine the advantages of solution-based enzymes, such as accessibility and ease of orientation, with the electrode, along with the benefits of immobilization on an electrode, including a reduction in the amount of enzyme required and, consequently, lower costs. This bioelectrode concept is based on the creation of a microcavity created by the assembly of two bucky papers containing one or more enzymes and possibly redox mediators.^167,168^

The operational stability of microbial BFCs is usually reported over a year, although longer studies are available.^110^ Despite the decline in electrical performance over time, the power output and waste treatment efficiency remain satisfactory, indicating that the lifespan of these systems can exceed one year. Typically, these systems exhibit an initial increase in power output during the first month due to the stabilization period of microbial colonies on the anode surface. However, over time, power output decreases due to nonelectrochemical active microorganisms,^111^ biofouling of membranes and cathodes,^111,114,117^ decreased oxygen diffusion,^117^ and variations in organic content and wastewater flow rate.^36,110^ The anode performance tends to be more stable than the cathode, with the decrease in cathode performance being the most critical factor for the long-term decline in BFC performance.^114,117^ The reduced cathodic performance is generally due to decreased oxygen diffusion through the electrode caused by biofilm formation or salt accumulation.^117^ Additionally, other factors, such as the organic content of the wastewater and the rate of wastewater flow into the system, also contribute to fluctuations in power output.^111,114,117^ Performance is usually reported in terms of volumetric power and energy, considering the electrolyte volume and flow rate. Improved standardization in these metrics would enhance comparability between different studies and researchers.^115,169,170^ Challenges for commercialization include low energy output and high production costs, particularly due to the use of cation exchange membranes.^115,169,171^ Full-scale demonstrations are limited, and material costs can be significant. Using inexpensive feedstock materials and advanced fabrication methods, like 3D printing, can reduce costs and while enhancing properties. Stacked configurations can enhance electricity generation.

As mentioned previously, the research published in Microbial RFC is in its early stages, so there are few studies detailing metrics and standardization. However, some studies indicate that a microbial RFC can operate for 29 charge–discharge cycles, achieving a Coulombic efficiency of approximately 99% and an energy efficiency of approximately 55%. These results indicate promising performance, although further studies are needed to confirm whether these are the best metrics achievable.^37^ Another study was able to produce current densities between 0.0200 and 0.0500 mA cm^–2^ and maximum power density of up to 0.0033 mW cm^–2^.^63^ Although these data seem promising, a new proof-of-concept study has managed to achieve current density above 40 mA cm^–2^ and power density above 10 mW cm^–2^, surpassing current results. According to the author, this improvement is attributed to the efficient electron transfer facilitated by the redox mediators used.^48^ Despite these promising results, microbial RFCs face challenges related to long-term stability.

Furthermore, a significant problem is biofouling on the cathode and membrane surfaces, which can impede ion transport and increase internal resistance, resulting in performance degradation over time. This issue is also observed in microbial BFCs, which present similar electrochemical principles.^172^ To mitigate these effects, the use of antifouling membranes and electrode materials, as well as operational modifications (such as periodic disconnection of the power supply), can be used.^173^ Furthermore, the selection of microbial communities is crucial to optimize the performance of microbial RFCs. Thus, ongoing research focusing on system optimization, material selection, and operational strategies will be fundamental to unlocking the full potential of microbial RFC technology.

## Bioelectrochemical Systems Roadmap

A roadmap for the future of BES, focusing on energy density, stability and validation for sustainable energy solutions, involves several key steps, as described in Figure 6. First, it requires identifying long-term stability challenges by conducting a comprehensive analysis of the issues faced by BES and pinpointing the main sources of degradation in biological activity, integrity, and energy efficiency. Next, specific evaluation methodologies tailored to the characteristics of BES need to be developed. These methodologies should consider the unique interaction between microbial communities and electrochemical materials, establishing performance metrics like specific energy, energy density, specific power, energy efficiency, and energy retention to assess stability. Improving BES efficiency and stability involves investing in research and development to address the identified challenges. This includes exploring new electrochemical, microbial, and enzymatic materials to optimize BES performance under prolonged operational conditions. Establishing a validation protocol for BES is crucial. This protocol should encompass standardized testing procedures, performance metrics, and reliability criteria, incorporating innovative assessment tools such as the bio-Ragone plot specific to BES (Figure 5) to provide insights into system performance and potential applications.

Improving BES efficiency and stability involves investing in research and development to address the identified challenges. This includes exploring new electrochemical, microbial, and enzymatic materials to optimize BES performance under prolonged operational conditions. Establishing a validation protocol for BES is crucial. This protocol should encompass standardized testing procedures, performance metrics, and reliability criteria, incorporating innovative assessment tools such as the bio-Ragone plot specific to BES (Figure 5) to provide insights into system performance and potential applications.

**Figure 6:**
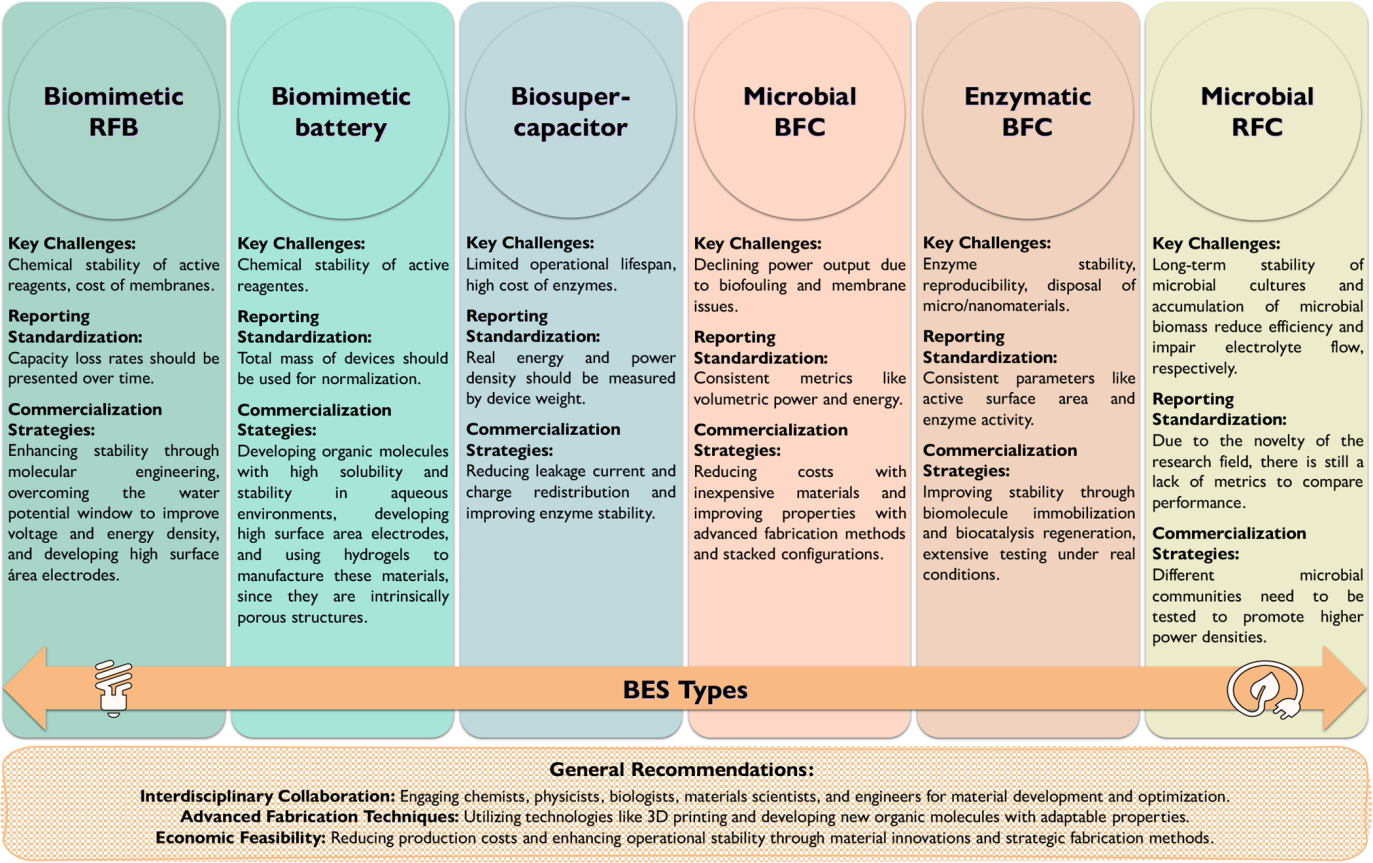
BES roadmap: key challenges, reporting standardization and commercialization strategies for different BES types.

Interdisciplinary collaboration and funding initiatives are necessary for advancing BES. Establishing research consortia and funding initiatives dedicated to BES encourages collaboration among researchers, industry partners, and regulatory bodies, promoting knowledge exchange across different disciplines. Standardizing testing protocols and benchmarking involve collaborative efforts with regulatory bodies and industrial organizations. This facilitates comparison among different systems and technologies, promoting transparency and replicability of research results through data-sharing practices. Technological advancements and scaling-up of BES require continued investment in research and development. This aims to enhance efficiency, reliability, and scalability, exploring opportunities for commercial and industrial applications in sectors such as wastewater treatment, renewable energy generation, and energy storage. Finally, education and awareness initiatives are essential. Promoting public education on the benefits and applications of BES as sustainable energy solutions while fostering awareness of the importance of stability and validation ensures widespread acceptance and adoption of BES technologies.

## Innovative Approaches in Bioelectrochemical Systems

Emerging approaches within BES include advanced biomimetic RFB, microbial electrochemical snorkels, hybrid enzymatic-microbial BFC, and disposable paper-based BFC. These innovations represent the frontier of BES research, where biomimetic RFB utilize synthetic molecules inspired by biological systems to enhance energy storage efficiency and stability. They can also adapt new RFB techniques for biomimetic RFBs, creating a pH difference between the two battery electrolytes that permits voltages to surpass the thermodynamic water splitting window.^81^ A promising alternative is to combine biobatteries with BFCs, as is the case with microbial BFCs. However, the idea is also to miniaturize the system, using disposable biomimetic batteries to power devices that require only minimal amounts of energy for a short period of time.^174^ Enzymatic cascades can be used to achieve high energy densities in enzymatic BFCs through deep/complete oxidation of fuels.^45^ In addition, increasing enzyme activity, facilitating electron transfer, using nanomaterials, and developing more efficient enzyme-electrode interfaces can be used to increase the power density of enzymatic BFCs.^45^ In terms of electrodes, fabric materials have been increasingly used in enzymatic BFCs, which are favorable for wearable electronic devices due to their flexibility.^46^ Several approaches are being used to improve the stability of enzymatic BFCs, including various enzyme immobilization procedures, modification of enzyme properties, development of protective matrices, and the use of enzymes that display the microbial surface properties.^45^

Stability is another critical enzyme feature. Operational conditions in enzymatic BFCs often involve prolonged exposure to the electrolyte, which can denature enzymes or contribute to enzyme leaching, reducing the enzymatic BFCs long-term operational stability. The entrapment of redox enzymes in gels has been demonstrated to be a promising strategy to overcome this issue.^120,175^ In cases where enzymatic electrode performance is limited by substrate mass transfer, as occurs in O_2_-biocathodes, the use of gas-diffusion electrodes has played a significant role. It is well-known that the use of gas diffusion electrodes (GDEs) is extremely attractive, because they allow freely gas permeability through a hydrophobic layer to reach the enzyme-based catalytic layer, leading to high reduction currents.^175,176^

Microbial electrochemical snorkels explore new designs to improve electron transfer between microbial communities and electrodes, potentially increasing power output and efficiency in microbial BFCs.^177^ Another approach to improving the performance of microbial BFCs is to use organic semiconductors to tune the interface between microbial systems and external electrodes.^47^ Hybrid enzymatic-microbial BFCs combine the catalytic properties of enzymes and the metabolic capabilities of microbes to create more versatile and efficient energy conversion systems. Considering that miniature conventional batteries are complex, expensive, and environmentally unfriendly to collect and recycle, thus constituting a significant source of pollution, enzymatic BFCs based on paper, proteins, and carbon constitute a green energy solution for the next generation of smart and sustainable electronics. Their industrial development is currently leading to thin, light and flexible prototypes that are disposable, recyclable, environmentally friendly and economically viable.^178,179^

Another innovative use of BES is the simulation of the Haber-Bosch process, which produces ammonia from nitrogen and hydrogen.^180,181^ Still widely employed today, this process revolutionized agriculture but now accounts for about 1% of global energy consumption due to the high pressures and temperatures required to drive the chemical reactions. One approach using BES to promote ammonia synthesis consists in a fuel cell that was used nitrogenase to reduce nitrogen (biocathode), hydrogenase to oxidase hydrogen (bioanode), and methyl viologen was used as electron mediator for both processes.^180^ It is also noteworthy that the H_2_/N_2_ BFC produces ammonia and generates electrical energy at the same time, instead of consuming huge amounts of energy as in the conventional Haber-Bosch process. Moreover, nitrogenases have been used in the electrosynthesis of value-added products.^182^ For instance, an enzymatic cascade was developed by utilizing nitrogenase, diaphorase, and alanine dehydrogenase to electrochemically drive transaminase far from its reactant favored equilibrium to produce chiral amines.^183^ Furthermore, cascade bioelectrocatalysis can be used in various other approaches, including CO_2_ fixation, high-value-added product formation, sustainable energy sources via deep oxidation, and cascaded bioelectrochemical reactions.^184^

Innovation in BES has significantly improved their performance. Continued improvements at these levels require cross-disciplinary collaborations and the integration of disparate fields for improvements beyond incremental. One emerging area of collaboration that has significantly enhanced current production from bioanodes is the deployment of conductive materials commonly used in the battery field for these electrodes. For example, the integration of ion- and electron-conductive polymers for carbon electrode surfaces has been repported.^185^ More than five times the current output was generated when *S. oneidensis* was grown on electrodes modified with ion- and electron-conductive P3HT-Imidazolium polymers as compared to either bare carbon or P3HT polymers alone.^186^ This improvement was found to be due to a change in the thermodynamics of electron transfer from flavins to the electrode surface. Instead of a conventional two-step electron transfer for the complete oxidation of the flavin at the electrode surface, in the presence of the polymer, a concerted, single-electron transfer is observed, significantly improving microbial electrochemical technologies. These improvements were further found to occur in the presence of ion-conductive imidazolium groups, even in the absence of electron-conductive polymers.^187^ Additional studies of electron transfer between small-molecule mediators and an electrode were conducted in ionic liquids where the cation was imidazolium.^188^ Similar changes in the electron transfer were observed there as well. Thus, critically, the integration of materials from next-generation batteries into BES has significantly improved BES performance.

Promising strategies for achieving high performance and commercial viability of BES involve biotechnological, synthetic, and material science aspects, such as the development of redox enzymes displayed on microbial surfaces,^189^ engineered living materials,^190^ nature-inspired materials,^191^ and artificial enzymes^192^ for selective catalysis and interactions. These innovations can significantly enhance stability and substrate specificity and reduce costs related to the obtention of the active materials. Similarly, synthetic biology is increasingly deployed to improve the biological components of BES. Studies of electron transfer and respiratory pathways in *S. oneidensis* have enabled the tunable current production from these microbes.^187^ Current production was significantly increased when the competitive hydrogen evolution respiratory pathway was knocked out of the cells. Similarly, the integration of the electron transfer-proficient Mtr pathway into *E. coli* has enabled these microbes to perform direct electron transfer.^193^ As is evident just from these two examples, synthetic biology is extensively used throughout the bioelectrochemical field to boost efficiency. Notably, however, is its application for improving enzymatic systems through integration with whole-cell systems. Recently, the integration of surface-expressed of enzymes on *E. coli* with sustainability-based catalysis proven promising for next-generation systems. As described in previous sections, many enzymes suffer from instability that limits their utility without significant modification with polymers or other stabilization approaches. Through surface display, improved protein stability, decreased cost and processing intensity, and higher activity are all observed. Although this approach has had limited applications in energy or catalysis to-date, its application in environmental contaminant monitoring and degradation highlights its potential in this field.

Together, these technologies enhance the scalability and long-term stability of BES, laying a solid foundation for their practical deployment in energy generation. While still in the experimental stage, these emerging approaches hold considerable promise for advancing energy storage and conversion, with the potential to significantly broaden the functionality and real-world applications of BES across diverse sectors.

## Pathways and Perspectives for BES Development

Beginning with a comprehensive review of the current literature on BES, it becomes evident that a thorough comparison of system performance is often lacking. Therefore, a detailed analysis of performance metrics for the BES, as documented in the literature survey, is presented. This analysis reveals that those variations in specific energy and specific power across different BES primarily stem from differences in electrode materials and system configurations. To establish a standardized benchmark, a BES configuration consisting of commonly used electrode materials and minimal additional components is introduced. Specifically, a BES with carbon-based anodes, microbial catalysts, and commonly employed cathode materials is considered. By intentionally avoiding complex modifications, these baseline BES configurations serve as fundamental references for comparison and evaluation. Utilizing performance metrics such as power density and energy efficiency, a direct comparison of different BES configurations and operating conditions is enabled relative to the baseline systems. This comparative analysis highlights areas of improvement and guides future research directions in BES development. Our analysis underscores the need for ongoing research to enhance the performance and scalability of BES technologies. Key research targets identified include maximizing power output, improving energy efficiency, optimizing electrode materials, and enhancing system stability.

Leveraging fundamental principles governing BES performance, a pathway toward more efficient and reliable bioelectrochemical systems is delineated. This pathway outlines specific targets, including achieving higher power densities, optimizing electron transfer kinetics, and maximizing substrate utilization rates to propel BES technology toward widespread adoption and integration into sustainable energy solutions. To advance knowledge and practical application of BES in sustainable energy solutions, it is crucial to investigate fundamental questions. First, it is necessary investigate the factors that affect the long-term stability of BES. Long-term stability is influenced by a range of variables, including electrode materials, operating conditions, and microbial communities present in the systems. Additionally, understanding the typical lifespan of BES and identifying possible outliers with exceptionally long lifespans is essential. This understanding is critical for assessing the economic viability and potential impact of BES.

Another important aspect is to moderate the influence of temperature on BES performance, as enzymatic and microbial activities are highly temperature dependent. This parameter can be extremely limiting for potential industrial developments of BES. However, it should be noted that this issue can prove to be an advantage in the case of implanted BFC, which, by definition, are exposed to a constant physiological temperature of 37 °C. Research will focus on selecting or engineering microorganisms that can thrive at higher or lower temperatures or designing enzymes with mutations that increase their thermal stability. This can be achieved through directed evolution or rational design methods.

Another challenge concerns the biocompatibility of BES. Ensuring the sterility of implantable BES is crucial for preventing microbial contamination and the subsequent disease transmission. Disinfection and sterilization of implantable BES are thus an important issue for the credibility of the potential applications envisioned.^194^ However, this problem is challenging to solve due to the fragility of biocatalysts with conventional treatments such as autoclaving, ethylene oxide sterilization or chemical sterilization.

Similarly, it is important to inquire about the current state of standardization in reporting BES results and how it can be improved to facilitate better comparability among researchers. The lack of standardized reporting protocols is a significant challenge in the field, and addressing this issue is essential for advancing the collective understanding of BES technology. Investigating the limiting factors and costs associated with BES that may hinder their commercialization is crucial. This includes exploring the economic feasibility of BES and identifying potential barriers to their widespread adoption. Lastly, it is important to explore how we can optimize the performance of BES to improve their long-term stability and commercial viability. This question emphasizes the practical importance of BES research and highlights the need for multifaceted approaches that incorporate advancements in materials science, system design, and operational strategies. Addressing these fundamental questions can significantly advance the development and application of bioelectrochemical systems as sustainable energy solutions.

## Supplementary Material



## Notes

Additional details, including numerical data and explanation of the choice of devices for the bio-Ragone plot, as well as the keywords searched indicating the distribution of document types and Web of Science categories (PDF)
