# Supplementary material for: Bioelectrochemical Systems: Prioritizing Energy Density, Long-Term Stability, and Validation
Source: ACS Energy Lett. 2025 Aug 20;10(9):4470–90. doi: 10.1021/acsenergylett.5c01678 (PMC12442509; doi:10.1021/acsenergylett.5c01678)
Supplement: Supplementary file 1 [file nz5c01678_si_001.pdf]

SUPPORTING INFORMATION

Bioelectrochemical systems: prioritizing energy density, long-term stability and validation

Luana C. I. Faria,[1] Steffane Q. Nascimento,[1] Filipe C. D A. Lima,[2] Graziela C. Sedenho,[1] Thiago Bertaglia,[1] Rodrigo M. Iost,[1,3] João C. P. de Souza,[4] Senentxu Lanceros-Méndez,[5,6,7] Shelley D. Minteer,[8,9] Serge Cosnier,[10,11] Ariel Furst[12] and Frank N. Crespilho[1*]

[1] São Carlos Institute of Chemistry, University of São Paulo, 13560-590, São Carlos, SP, Brazil.

[2] Federal Institute of Education, Science and Technology of São Paulo, 15991-502, Matão, SP, Brazil.

[3] Department of Fundamental Chemistry, Institute of Chemistry, University of Sao Paulo, 05508-000, Butantã, SP, Brazil.

[4] Faculty of Sciences, São Paulo State University, 17033-360, Bauru, SP, Brazil.

[5] Physics Centre of Minho and Porto, Universities (CF-UM-UP) and Laboratory of Physics for Materials and Emergent Technologies, LapMET, University of Minho, 4710-057 Braga, Portugal.

[6] BCMaterials, Basque Center for Materials, Applications and Nanostructures, 48940 Leioa, Spain.

[7] Ikerbasque, Basque Foundation for Science, 48009 Bilbao, Spain.

[8] Department of Chemistry, Missouri University of Science and Technology, 65409-6518, Rolla, Missouri, United States.

[9] Kummer Institute Center for Resource Sustainability, Missouri University of Science and Technology, 65409-6518, Rolla, Missouri, United States.

[10] Center for Organic and Nanohybrid Electronics, Silesian University of Technology, Konarskiego 22B, 44-100 Gliwice, Poland.

[11] Department de Chimie´Moleculaire, CNRS UMR-5250, Université Grenoble Alpes, F-38000 Grenoble, France.

[12] Department of Chemical Engineering, Massachusetts Institute of Technology, 02139, Cambridge, MA, United States.

[*]Corresponding author

E-mail address: frankcrespilho@iqsc.usp.br (F.N.Crespilho)

Contents

1. GENERAL 3
2. CHOOSING DEVICES FOR THE BIO-RAGONE PLOT 5
3. BIOMIMETIC REDOX FLOW BATTERY CALCULATION 7
4. BIOMIMETIC BATTERY CALCULATION 8
5. BIOSUPERCAPACITOR CALCULATION 9
6. ENZYMATIC BIOFUEL CELL CALCULATION 11
7. MICROBIAL BIOFUEL CELL CALCULATION 12
8. MICROBIAL REDOX FLOW CELL CALCULATION 15
9. KEYWORDS 16
   8.1. Biomimetic redox flow batteries 16
   8.2. Biomimetic battery 16
   8.3. Biosupercapacitor 17
   8.4. Microbial biofuel cell 17
   8.5. Enzymatic biofuel cell 18
   8.6. Microbial redox flow cell 18
9. REFERENCES 19

1. GENERAL

The supporting information provides detailed experimental methodologies for various bioelectrochemical systems, including biomimetic redox flow batteries, biomimetic batteries, biocapacitors, microbial biofuel cells, enzyme biofuel cells, and microbial redox flow cells. Each section outlines specific calculations used to determine parameters such as power density, energy density, specific power, and specific energy. These calculations involve factors such as electrode area, electrolyte volume, battery capacity, voltage, and current. Standardization of units and normalization of values are emphasized to facilitate comparisons between different studies. Additionally, considerations for experimental variables affecting system performance, such as electrode materials, system configuration, and electrolyte flow rate, are discussed. Finally, the estimation of specific power and energy density is rationalized based on the aqueous nature of electrolytes in these bioelectrochemical systems.

The literature review conducted for this study, spanning from April to May 2024, involved in-depth scientific and technological exploration of key terms associated with energy generation and storage. Only original research articles and patents detailing the development of these biodevices were considered. The search for patents utilized the Patentscope database, which aggregates major patent databases worldwide, including INPI, Espacenet, and USPTO. Meanwhile, the Web of Science journal database was employed to source articles. English keywords such as Biocapacitor, Enzymatic Biofuel Cell, Microbial Biofuel Cell, Microbial Fuel Cell, Biomimetic Battery, and Biomimetic Redox Flow Battery were used for scientific prospecting. Searches were limited to the fields of "title, abstract, and keywords" within the last 5 years. Analysis in the Web of Science database included examining the yearly publication count and identifying the countries prominently contributing to this research. For technological prospecting in the Patentscope database, searches were confined to the fields of "title, summary, and keywords." Unlike scientific prospecting, no specific timeframe was defined. Instead, the study focused on gathering the total number of patents and analyzing their distribution across different regions. This comprehensive approach aimed to provide a holistic understanding of recent advancements and trends in biodevice research and development for energy generation and storage.

While numerous articles were available for consideration, our focus leaned towards prioritizing recent works and those presenting clear and concise information for readers. Additionally, articles that included detailed calculations were given precedence. This approach aimed to ensure that the selected literature would provide readers with the most up-to-date insights and comprehensible

data, facilitating their understanding of the topic at hand. By prioritizing recent and informative works, we aimed to offer the most relevant and valuable information available in the field of study.

For **Figure 3**, energy density data were sourced from commercial battery companies specializing in a variety of battery chemistries, including Nickel-zinc, Rechargeable alkaline, Lithium-titanate, Silver-oxide, Lithium manganese oxide, Lithium iron phosphate, Lead-acid (SLA), Alkaline, Lithium cobalt oxide, Lithium nickel cobalt aluminum oxide, Lithium nickel manganese cobalt oxide, Lithium-nickel cobalt aluminum oxide, Lithium-iron disulfide, Lithium-manganese dioxide, Zinc-air, and Lithium-carbon monofluoride. These data points were instrumental in providing a comprehensive overview of energy density across different battery types.

2. CHOOSING DEVICES FOR THE BIO-RAGONE PLOT

Bioinspiration and sustainability were the main criteria used in choosing the biobattery studies for discussion in this review article. In this context, all the selected studies report the use of phenazines (which act as adenosine A2B receptor modulator), alloxazine derivatives (essential for several cellular processes) and quinones (essential electron carriers for cellular respiration and photosynthesis) as electroactive elements for energy storage. However, biobatteries differ in their operating mechanism and composition, which may explain the variability obtained in the reported data. Factors such as the lack of studies on the diffusion of quinone-based supramolecular structures, the battery engineering process, the lower solubility of quinone compounds, among other factors, limit the energy density of biobatteries. We would like to emphasize that the choice of batteries with different operating principles is essential to compare the developed technology and point out its benefits and disadvantages to the scientific community, which is in line with the objective of our work. Furthermore, organic-based lithium-ion batteries (LIBs) and sodium-ion batteries (SIBs) were not chosen due to their low sustainability, as they generally rely on unsustainable organic solvents. In this sense, all the chosen studies use aqueous electrolytes or ionic liquids, which are less harmful.

In particular, the data for biosupercapacitors were extracted from the study "Biosupercapacitor with an enzymatic cascade at the anode working in a sucrose solution",[1] which presents a Ragone plot comprising ten distinct experimental data points, already expressed in standardized units of energy and power densities (Wh kg^{-1} and W kg^{-1}, respectively). This reference was selected due to its scientific significance and the methodological rigor in presenting comparable metrics across multiple devices. The study exemplifies recent advancements in biosupercapacitor design, notably through the implementation of a multienzymatic cascade at the anode, which enhances energy conversion efficiency. Importantly, the considerable variation in performance among devices of the same category, as observed in this work, underscores the profound influence of parameters such as electrode composition, enzymatic configuration, electrolyte formulation, and device architecture. These intrinsic variabilities highlight both the potential and the limitations of current biosupercapacitor technologies and reinforce the necessity of critically evaluating the factors that govern their performance.

Concerning the microbial BFC, we have selected the most recent and relevant works that reports unit cell results with power and energy values, which are required to construct Ragone plot. This was a challenge because many works do not report both performance parameters or do not provide enough data to calculate those. The significant performance variation among the selected works lies in the system dimensions and conditions. For example, a miniaturized microbial fuel cell reached higher volumetric power than larger setups. In addition, the conditions, such as wastewater

composition and temperature, affect the metabolic pathways and microbial proliferation, directly impacting the system performance. We have intentionally selected works with different microbial fuel cell designs and operating under diverse conditions to cover the variations and explicit the potential of those systems.

We conducted a meticulous selection of the most recent and relevant studies on enzymatic devices, with particular attention to kinetic, thermodynamic, and energy density performance. In this process, rigorous criteria were applied to ensure the proper consideration of essential experimental parameters, such as electrode surface area, active material loading, and system configuration. It is also acknowledged that significant variations in energy density can arise from factors such as electrode composition, surface chemistry, and operational conditions, all of which are critical for assessing the scalability of BES technologies. Our systematic and parameter-conscious approach ensures that the performance variations captured in this study are both representative of the broad landscape of BES technologies and sufficiently detailed to support meaningful comparative analyses.

For the microbial RFC studies, the main selection criteria were bioinspiration, technological novelty, and demonstrated system scalability, consistent with the overarching objective of this review to highlight sustainable and biologically integrated approaches to energy storage and conversion. Specifically, we prioritized studies that employ electroactive microorganisms in combination with soluble redox mediators, since these compounds play critical roles in natural electron transport chains and have shown promising performance in microbial RFC configurations. A further criterion was the availability of clear and standardized performance data, such as power and energy densities. We acknowledge that the relatively small number of microbial RFC studies currently available inherently limits the breadth of examples, especially when compared to more mature BES types. Nonetheless, we intentionally included works that span different operating conditions, and microbial consortia to illustrate the current landscape and the principal factors contributing to the observed performance variations.

We believe that providing this broad, yet representative snapshot aligns with the primary goal of this work: to critically assess the current status of each BES type and to highlight the areas where further development is needed to reduce performance variability and enhance scalability. We emphasize that this heterogeneity is not only a technical challenge but also an opportunity to understand how specific design choices impact performance of BES. This analysis is critical for establishing meaningful benchmarks across the field.

3. BIOMIMETIC REDOX FLOW BATTERY CALCULATION

For the articles that did not provide power data, we calculated it as follows:

$$Power\ (W\ L^{-1}) = \frac{Power\ density\ (W\ cm^{-2})\ x\ Area\ (cm^{2})}{Total\ volume\ (L)} \quad (Equation\ S1)$$

For articles that did not provide energy data, we calculated it as follows:

$$Energy\ (Wh\ L^{-1}) = Volumetric\ capacity\ (Ah\ L^{-1})\ x\ Voltage\ (V) \quad (Equation\ S2)$$

Or:

$$Energy\ (Wh\ L^{-1}) = \frac{Discharge\ capacity\ (Ah)\ x\ Voltage\ (V)}{Total\ volume\ (L)} \quad (Equation\ S3)$$

Briefly, power calculations (W L^{-1}) of the redox flow battery were calculated by multiplying the power density data (W cm^{-2}) by the electrode area (cm^{-2}) and then dividing by the total volume of redox species (L).

The energy calculation (Wh L^{-1}) was calculated in two different ways, according to the data provided by the articles. The first was by multiplying the volumetric capacity (Ah L^{-1}) and battery voltage (V). In the second way, when the capacity data was not presented, we multiplied the discharge capacity (Ah) by the battery voltage (V) and divided by the total volume of the electrolyte. Finally, the specific power (W kg^{-1}) and specific energy (Wh kg^{-1}) data were normalized considering the water density (1 kg L^{-1} at 25ºC). Since all electrolytes in redox flow batteries are aqueous, this estimate seems suitable.

Table S1 - Data used to prepare the Ragone plot of biomimetic redox flow batteries.

Type	Total volume / L	Area / cm^2	Power Density / mW cm^{-2}	Power / W Kg^{-1}	Discharge Capacity / A h	Volumetric capacity / Ah L^{-1}	Voltage / V	Energy / Wh kg^{-1}	REF
**Naphthoquinone**	0.045	5.0	264.0	29.3	-	5.32	0.92	4.89	[2]
**Phenazine**	0.047	5.0	94.0	10.0	0.0362	80.9	1.15	93.0	[3]
**Flavin**	80.0	25.0	160.0	0.05	-	5.03	0.96	4.83	[4]
**Phenazine**	0.03	5.0	140.0	23.3	-	2.80	1.70	4.76	[5]
**Quinone**	0.05	5.0	5.63	0.563	0.0333	-	0.72	0.48	[6]
**Alloxazine**	0.0143	5.0	350.0	123.0	-	108.0	1.20	130.0	[7]
**Phenazine**	0.047	5.0	103.0	11.0	0.0338	53.9	1.15	62.0	[8]
**Anthraquinone**	0.024	4.0	490.0	81.7	-	4.0	1.10	4.4	[9]
**Anthraquinone**	0.035	5.0	180.0	25.7	-	4.76	1.14	5.43	[10]

4. BIOMIMETIC BATTERY CALCULATION

When not provided in the text or graphics of the articles, specific energy and power values were calculated using the following equations:

$$Energy = Q_t \times V \quad (Equation\ S4)$$

$$Power = I \times V \quad (Equation\ S5)$$

Briefly, battery capacity (Q_t) and voltage values were multiplied to provide battery energy (typically in mWh g^{-1}). The next step was to calculate the amount of active compound in the battery, based on the "loading" values and electrode area. This value was used to estimate the battery's gross energy and subsequently used to calculate the Wh kg^{-1} energy values.

To calculate the power, the current values were estimated using the battery capacity and the discharge time provided by the galvanostatic discharge graph. In the case of a description of the current value drawn from the battery in the experimental section, this value was used. The specific power was then obtained by multiplying the open circuit voltage (OCV) and current values followed by dividing the estimate of active compound in the electrode, providing the values in W kg^{-1}.

Table S2 - Data used to prepare the Ragone plot of biomimetic batteries.

Type	Power / W Kg^{-1}	Energy / Wh kg^{-1}	REF
**Anthraquinone**	290.0	25.0	[11]
**Quinone**	2700.0	436.0	[12]
**Naphthoquinone**	240.0	49.0	[13]
**Quinone**	20000.0	100.0	[14]
**Quinone**	25.6	133.0	[15]
**Anthraquinone**	4.46	0.33	[16]

5. BIOSUPERCAPACITOR CALCULATION

Analyzing and comparing the power and energy of biosupercapacitors presents a significant challenge due to the variability in units utilized, as some values are given in geometrical units, such as W cm^{-2} and W h cm^{-2}. Consequently, without incorporating the material densities and volumetric specifications of biosupercapacitor devices, achieving standardization in calculations for power and energy becomes unfeasible. The ideal method for reporting these units in a Ragone plot is by using W kg^{-1} and W h kg^{-1}, respectively. For instance, Kizling et al.[1] reported data from a biosupercapacitor using conventional units for plotting the energy-power relationship of an energy storage material, device, or system, which are presented in Table S3. These values were extracted from Ragone plot reported by Kizling et al.[1]. Table S4 presents some values for biosupercapacitors by using no conventional units that brings more information about the power and energy densities of these devices.

Table S3 - Data used to prepare the Ragone plot of biosupercapacitors.

Type	Power / W kg^{-1}	Energy / Wh kg^{-1}	REF
**Bilirubin oxidase/cellobiose dehydrogenase**	11.0	3.78	17
**Glucose oxidase/laccase**	20.0	3.84	18
**Glucose dehydrogenase/bilirubin oxidase**	48.9	3.65	19
**Ceramic Microbial Fuel Cells – Supercapacitive mode**	106.0	3.52	20
**Laccase/fructose dehydrogenase**	145.0	3.28	21
**Glucoese dehydrogenase/bilirubin oxidase**	213.0	3.16	22
**Glucose, fructose and sucrose**	287.0	3.05	23
**Bilirubin oxidase**	461.0	2.95	24
**Implantable anti-biofouling biosupercapacitor**	1190.0	2.60	25
**D-fructose**	1540.0	2.03	26

Table S4 - Data in conventional units for biosupercapacitors.

Type	Power density	Energy density	REF
**Bilirubin oxidase/cellobiose dehydrogenase**	1.2 mW cm^{-2}	-	[17]
**Glucose oxidase/laccase**	16.0 mW	-	[18]
**Glucose dehydrogenase/bilirubin oxidase**	3.9 mW g^{-1}	-	[19]
**Ceramic Microbial Fuel Cells – Supercapacitive mode**	27.4 W m^{-3}	-	[20]
**Laccase/fructose dehydrogenase**	2.0 mW	-	[21]
**Glucoese dehydrogenase/bilirubin oxidase**	0.87 mW cm^{-2}	-	[22]
**Glucose, fructose and sucrose**	1.3 mW cm^{-2}	-	[23]
**Bilirubin oxidase**	6.0 mW cm^{-2}	-	[24]
**Implantable anti-biofouling biosupercapacitor**	25.52 mW cm^{-2}	274.0 μWh cm^{-2}	[25]
**D-fructose**	3.82 ± 0.01 mW cm^{-2} mM^{-1}	-	[26]

6. ENZYMATIC BIOFUEL CELL CALCULATION

The power calculation of the enzymatic biofuel cells was realized by dividing the power density data by the electrode area (W cm^{-2}) and the volumetric power density was obtained by dividing the power density area by the volume of the electrolyte solution (W m^{-3}). The volumetric energy density varied with each experimental setup and was specifically considered for the duration of the cell's power density operation.

Table S5 - Data used to prepare the Ragone plot of enzymatic biofuel cells.

Type	Total volume / L	Area / cm^2	T / ºC	Power Density / mW cm^{-2}	Power / W kg^{-1}	Energy / Wh kg^{-1}	REF
**Glucose/dioxygen – Implantable in Rat**	0.02	10.0	25.0	0.095	0.00048	0.0012	27
**Trehalase|glucose oxidade and bilirubin oxidase dioxygenen – Implantable in insect**	0.000007	0.071	25.0	0.055	0.00785	0.0157	28
**Glucose oxidase/laccase – Miniaturized, pH 5**	0.00001	0.0044	23.0 – 37.0	0.137	0.00137	0.032	29
**Glucose oxidase/laccase – Porous carbon, pH 7**	0.00039	1.30	25.0	1.25	3.21	0.032	30
**Glucose/CO_2 with microorganism *Rhodoferax ferrireducens* – Mediatorless microbial fuel cells**	0.21	65.9	25.0	-	-	-	31
**H_2/O_2 and O_2-sensitive hydrogenase – Viologen hydrogel, pH 7**	0.00005	0.5	40.0	0.178	0.0356	0.03601	32
**Glucose/oxygen – CNT EBFC, pH 7.2**	0.05	75000.0	37.0	0.74	0.00015	0.022	33
**Glucose/oxygen – biscrolled carbon nanotube**	0.05	0.0075	37.0	2.18	0.0436	0.403	34
**hydrogenase/polymer – pH 7.4**	0.01	0.07	-	0.53	0.053	0.053	35
**Glucose oxidase – LbL Cotton GOx, pH 7.4**	0.05	0.0314	36.5	3.70	0.074	0.012	36

7. MICROBIAL BIOFUEL CELL CALCULATION

The electrochemical performance of microbial biofuel cells is reported using different parameters and units, such as current density and power density, in which the values are normalized by the electrode area; volumetric power and volumetric energy, in which the values are normalized by the electrolyte volume. To standard the units and build the Ragone plot, when density values had been reported by authors, they were multiplied by the electrode area and divided by the electrolyte volume to have the volumetric values (e.g. W m^{-3})[37–39]. When only power (or energy) value had been reported, the energy (or power) was calculated by equations provided by authors [38,40–45]. In all these cases, the electrolyte flow rate was considered. In some cases, power (in watt) values were calculated from reported current and voltage values by multiplying them, as watt (W) is ampere (A) × volt (V) [39,46]. Finally, the specific power (W kg^{-1}) and energy (Wh kg^{-1}) were estimated considering the water density (1 kg L^{-1} at 25 ^{o}C) and the reported volumetric values. This estimation is reasonable as all electrolytes are aqueous in microbial biofuel cells.

It is evident that specific power and energy density of the microbial biofuel cells are affected by the extracellular electron transfer kinetic of the microorganisms involved. However, other experimental factors also play a significant role in the electrical output of those systems, such as the cathode material employed, system configuration, electrolyte flow rate, and fuel concentration. The latter is related to the wastewater features and is proportional to the oxygen demand and organic carbon content.

Table S6 - Data used to prepare the Ragone plot of microbial biofuel cells.

Type	Total volume / L	Area / cm^2	T / °C	Power Density / $mW\ cm^{-2}$	Power / $W\ kg^{-1}$	Energy / $Wh\ kg^{-1}$	REF
**Wastewater treatment**	1000.0	-	11.5-21.0	-	0.0003 / 0.0012 [a]	0.015 / 0.06	40
**Wastewater treatment**	1000.0	-	-	-	0.0335 [b]	0.033 / 0.005	47
**Wastewater treatment**	72.0	-	-	-	0.0256 / 0.0509	2.51 / 4.99 [c]	37
**Wastewater treatment**	45.0	2480.0	12.0–30.0	0.0073 / 0.01	0.000545 / 0.000583 [d]	0.007 / 0.025	41
**Wastewater treatment**	90.0	600.0	~ 25.0	0.0138 / 0.0181	0.000092 / 0.000121	0.056 / 0.097	48
**Wastewater treatment**	200.0	-	~7.0-27.0	200 mW (not normalized)	0.00053 / 0.00072 [e]	0.003 / 0.009	46
**Biomass**	0.000001	-	40.0 ± 3	-	10.5 / 11.9	0.0058 [f]	42
**Wastewater treatment**	110.0	734.0	-	0.0032 / 0.0042	0.00031 / 0.000414	0.00145 [f]	43
**Wastewater treatment**	125.0	-	10.0–12.0	-	0.008	0.686 [f]	44
**Wastewater treatment**	850.0	-	-	0.0043	0.000541 [c]	0.0134 [f]	38
**Ethanol MFC**‡	0.027	1.5	40.0	0.00000707	0.000393 [c]	0.006 [g]	39

‡ Cooperative microbial FC, where yeast and enzyme (alcohol dehydrogenase) were used.

[a] Calculated based on the equation provided by authors:[40]

$$NER_V = \frac{\underline{P}}{q_{An}} \quad (Equation\ S6)$$

where NER_v is equivalent to the volumetric energy (kWh m^{-3}), $\underline{P}$ is the average power (kW), and q_{An} is the anolyte flow rate ($m^3\ h^{-1}$). For calculation of volumetric power, it was considered MFC volume equals to 1000 L (1 m^3), as provided by authors.

[b] Authors reported unstable maximum power density within the range of 7-60 W m^{-3}.[47] Therefore, it was calculated the average value as 33.5 W m^{-3}.

[c] The volumetric power values were calculated based on the reported power density values, according to Equation S7:

$$volumetric\ density\ (W\ m^{-3}) = \frac{power\ density\ (W\ m^{-2}) \times electrode\ area\ (m^2)}{electrolyte\ volume\ (m^3)} \quad (Equation\ S7)$$

[d] Calculated based on the adapted equation provided by authors:[41]

$$NER_V = \frac{\underline{P} \times t}{V_{MFC}} \quad (Equation\ S8)$$

where NER_v is equivalent to the volumetric energy (kWh m^{-3}), $\underline{P}$ is the average power (kW), and V_{MFC} is the liquid volume of the MFC (L), and *t* is the period of a batch cycle (h). For calculation of volumetric power, it was considered MFC volume equals to 45 L (0.045 m^3), as provided by authors.

[e] Volumetric power values were calculated based on the data from Table 1 and Figure 6b of Ref.[46] Volumetric current values were multiplied by the mean MFC voltage (1V) to have the volumetric power values at different conditions.

[f] Volumetric energy values were calculated based on Equation S9:[12]

$$volumetric\ energy\ (\,Wh\,m^{-3}) = \frac{power\ (W)}{flow\ rate\ (m^3\ s^{-1})} \times \frac{1\,h}{3600\ (s)} \quad (Equation\ S9)$$

For the cases in which authors reported volumetric power value, the power was calculated by multiplying the volumetric power by the electrolyte volume.

[g] Calculated from voltage-time curve recorded at 10 μA cm^{-2} during 24 h.[39] The volumetric energy was calculated by integrating this curve and multiplying by the drained current (10 μA cm^{-2} × 1.5 cm^2), and then normalizing by the electrolyte volume (27 mL).

8. MICROBIAL REDOX FLOW CELL CALCULATION

In an overview, the microbial redox flow cell's power calculations (W L^{-1}) were determined by multiplying the power density data (W cm^{-2}) by the electrode area (cm^{-2}) and dividing the result by the total volume of chambers (L). Except for the Molenaar paper [49] which indicates that power is calculated by normalizing the bioelectrode volume (33 cm^3) or membrane surface area (22 cm^2).

Based on the information provided by the articles, two methods were used to calculate the energy density (Wh L^{-1}). Multiplying the volumetric capacity (Ah L^{-1}) by the battery voltage (V) was the initially employed method. The second method involved multiplying the discharge capacity (Ah) by the battery voltage (V) and dividing the result by the total volume, in the case that volumetric capacity data was not provided. Finally, considering the water density (1 kg L^{-1} at 25ºC), the specific power (W kg^{-1}) and specific energy (Wh kg^{-1}) measurements were standardized. This estimate seems reasonable because the electrolytes in all microbial redox flow cells are aqueous.

Table S7 - Data used to prepare the Ragone plot of microbial redox flow cells.

Type	Total volume / L	Area / cm^2	Power Density / W cm^{-2}	Power / W kg^{-1}	Volumetric capacity / Ah L^{-1}	Voltage / V	Energy / Wh kg^{-1}	REF
**Geobacter sulfurreducens**	0.1	9.0	0.00032	0.0288	0.01750	0.62	0.0109	[50]
**Ferrous-oxidizing bacteria**	0.24	6.25	0.00034	0.00891	0.06	0.32	0.0195	[51]
**CO_2-reducing charging cell and acetate-oxidizing discharging cell**	0.033	22.0	0.00030	0.20	-	0.60	0.1	[49]

9. KEYWORDS

8.1. Biomimetic redox flow batteries

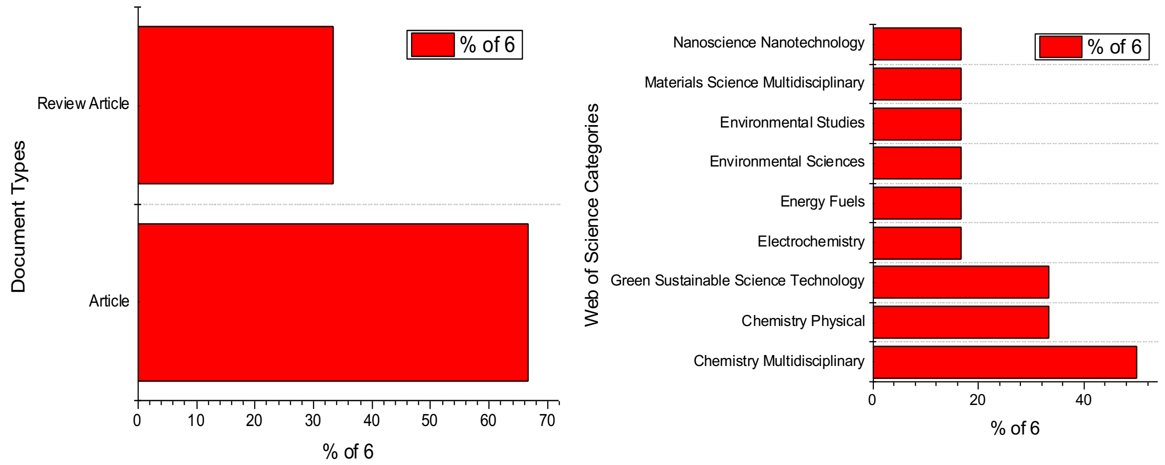

Figure S1 - Distribution of document types and Web of Science categories for the Biomimetic redox flow batteries. The left panel shows the percentage distribution of review articles and research articles. The right panel illustrates the percentage distribution across Web of Science categories.

8.2. Biomimetic battery

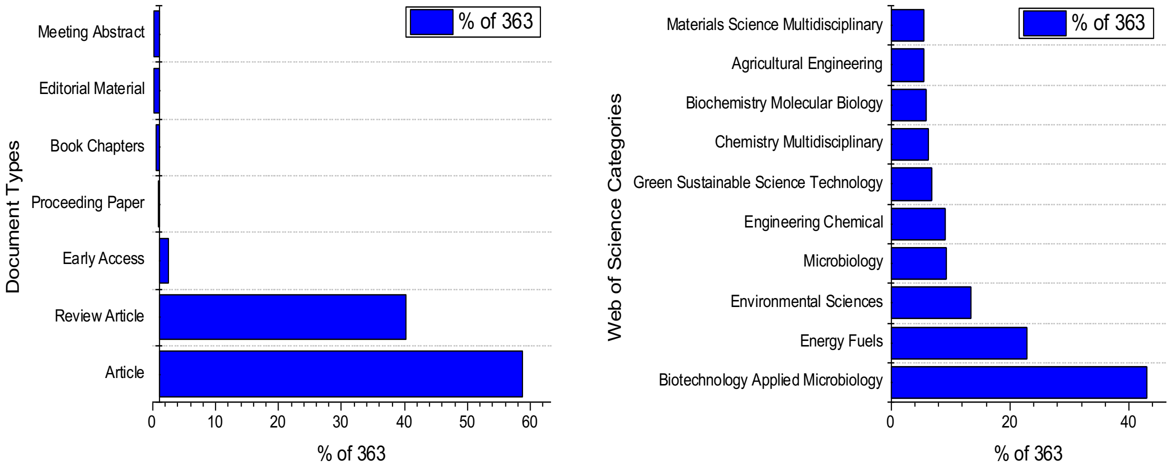

Figure S2 - Distribution of document types and Web of Science categories for biomimetic batteries. The left panel shows the percentage distribution of different type of scientific publications. The right panel illustrates the percentage distribution across Web of Science categories.

8.3. Biosupercapacitor

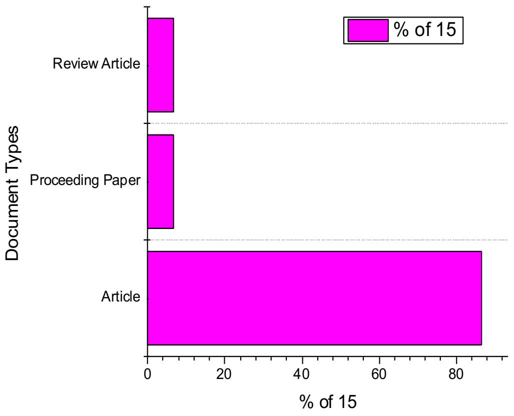

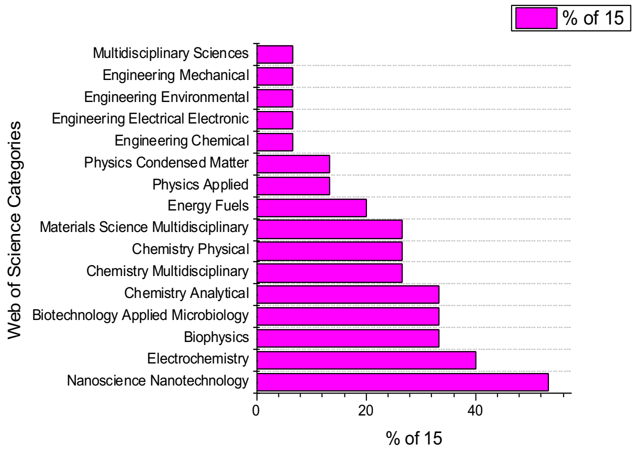

Figure S3 - Distribution of document types and Web of Science categories for biosupercapacitor. The left panel shows the percentage distribution of review articles, proceeding paper and research articles. The right panel illustrates the percentage distribution across Web of Science categories.

8.4. Microbial biofuel cell

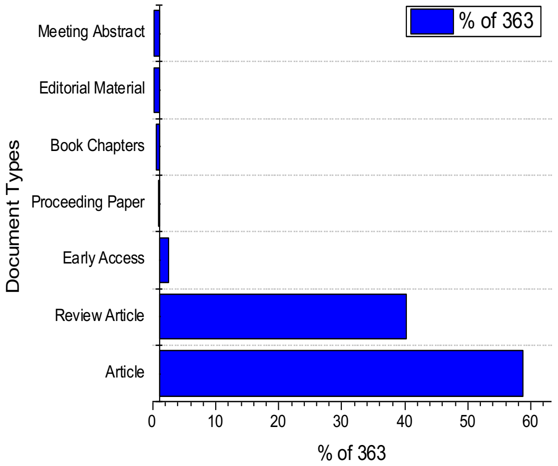

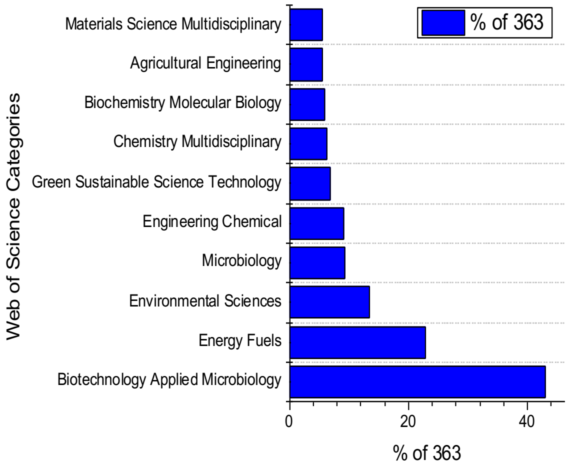

Figure S4 - Distribution of document types and Web of Science categories for microbial biofuel cell. The left panel shows the percentage distribution on document types. The right panel illustrates the percentage distribution across Web of Science categories.

8.5.Enzymatic biofuel cell

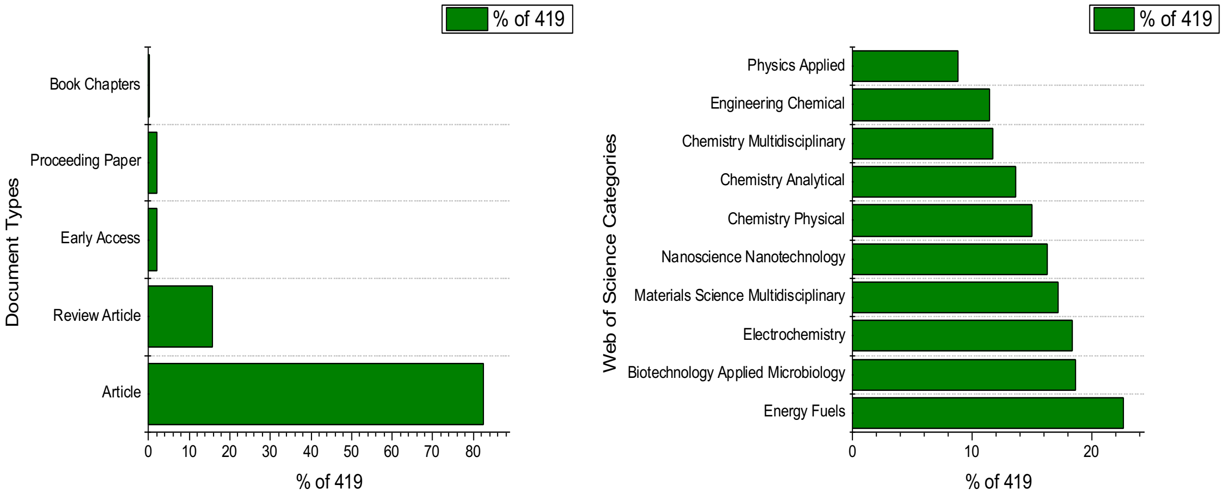

Figure S5 - Distribution of document types and Web of Science categories for studies analyzed in the context of enzymatic biofuel cell. The left panel shows the percentage distribution of various document types, with research articles constituting the majority. The right panel displays the percentage distribution across different Web of Science categories.

8.6.Microbial redox flow cell

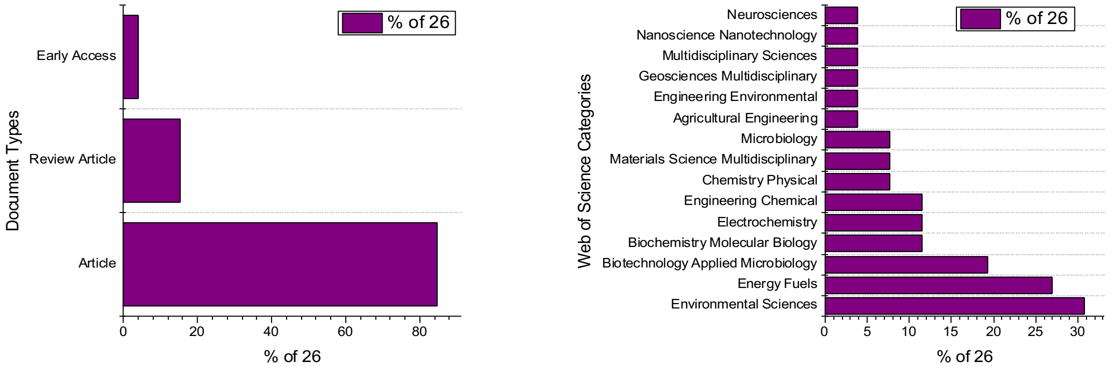

Figure S6 - Distribution of document types and Web of Science categories for research related to microbial redox flow cells. The left panel shows the percentage distribution of document types, indicating a predominance of research articles. The right panel illustrates the percentage distribution across some Web of Science categories.

9. REFERENCES

(1) Kizling, M.; Dzwonek, M.; Więckowska, A.; Stolarczyk, K.; Bilewicz, R. Biosupercapacitor with an Enzymatic Cascade at the Anode Working in a Sucrose Solution. *Biosensors and Bioelectronics* **2021**, *186*, 113248. https://doi.org/10.1016/j.bios.2021.113248.

(2) Liu, Y.; Zhang, P.; Wu, Z.; Ding, G.; Song, X.; Ma, J.; Wang, W.; Wang, X.-Z.; Jin, Z. Biomimetic Naphthoquinone Zwitterion Derivative with Water-Solubilizing Amino Acid Side Chain for High-Stability Aqueous Redox Flow Batteries. *ACS Energy Letters* **2024**, *9* (2), 586–593. https://doi.org/10.1021/acsenergylett.3c02530.

(3) Xu, J.; Pang, S.; Wang, X.; Wang, P.; Ji, Y. Ultrastable Aqueous Phenazine Flow Batteries with High Capacity Operated at Elevated Temperatures. *Joule* **2021**, *5* (9), 2437–2449. https://doi.org/10.1016/j.joule.2021.06.019.

(4) Orita, A.; Verde, M. G.; Sakai, M.; Meng, Y. S. A Biomimetic Redox Flow Battery Based on Flavin Mononucleotide. *Nature Communications* **2016**, *7* (1), 13230. https://doi.org/10.1038/ncomms13230.

(5) Xi, D.; Alfaraidi, A. M.; Gao, J.; Cochard, T.; Faria, L. C. I.; Yang, Z.; George, T. Y.; Wang, T.; Gordon, R. G.; Liu, R. Y.; Aziz, M. J. Mild PH-Decoupling Aqueous Flow Battery with Practical PH Recovery. *Nature Energy* **2024**. https://doi.org/10.1038/s41560-024-01474-1.

(6) Faria, L. C. I.; Sedenho, G. C.; Bertaglia, T.; Macedo, L. J. A.; Crespilho, F. N. A Comparative Study of Chemically Oxidized Carbon Cloth and Thermally Treated Carbon Paper Electrodes Applied on Aqueous Organic Redox Flow Batteries. *Electrochimica Acta* **2024**, *485*, 144086. https://doi.org/10.1016/j.electacta.2024.144086.

(7) Lin, K.; Gómez-Bombarelli, R.; Beh, E. S.; Tong, L.; Chen, Q.; Valle, A.; Aspuru-Guzik, A.; Aziz, M. J.; Gordon, R. G. A Redox-Flow Battery with an Alloxazine-Based Organic Electrolyte. *Nature Energy* **2016**, *1* (9), 16102. https://doi.org/10.1038/nenergy.2016.102.

(8) Pang, S.; Wang, X.; Wang, P.; Ji, Y. Biomimetic Amino Acid Functionalized Phenazine Flow Batteries with Long Lifetime at Near-Neutral PH. *Angewandte Chemie International Edition* **2021**, *60* (10), 5289–5298. https://doi.org/10.1002/anie.202014610.

(9) Liu, Y.; Lu, S.; Chen, S.; Wang, H.; Zhang, J.; Xiang, Y. A Sustainable Redox Flow Battery with Alizarin-Based Aqueous Organic Electrolyte. *ACS Applied Energy Materials* **2019**, *2* (4), 2469–2474. https://doi.org/10.1021/acsaem.8b01512.

(10) Wu, M.; Bahari, M.; Jing, Y.; Amini, K.; Fell, E. M.; George, T. Y.; Gordon, R. G.; Aziz, M. J. Highly Stable, Low Redox Potential Quinone for Aqueous Flow Batteries**. *Batteries & Supercaps* **2022**, *5* (6). https://doi.org/10.1002/batt.202200009.

(11) Xu, Y.; Zheng, Y.; Wang, C.; Chen, Q. An All-Organic Aqueous Battery Powered by Adsorbed Quinone. *ACS Applied Materials & Interfaces* **2019**, *11* (26), 23222–23228. https://doi.org/10.1021/acsami.9b05159.

(12) Li, Y.; Lu, Y.; Ni, Y.; Zheng, S.; Yan, Z.; Zhang, K.; Zhao, Q.; Chen, J. Quinone Electrodes for Alkali–Acid Hybrid Batteries. *Journal of the American Chemical Society* **2022**, *144* (18), 8066–8072. https://doi.org/10.1021/jacs.2c00296.

(13) Wang, H.; Emanuelsson, R.; Karlsson, C.; Jannasch, P.; Strømme, M.; Sjödin, M. Rocking-Chair Proton Batteries with Conducting Redox Polymer Active Materials and Protic Ionic Liquid Electrolytes. *ACS Applied Materials & Interfaces* **2021**, *13* (16), 19099–19108. https://doi.org/10.1021/acsami.1c01353.

(14) Yang, X.; Ni, Y.; Lu, Y.; Zhang, Q.; Hou, J.; Yang, G.; Liu, X.; Xie, W.; Yan, Z.; Zhao, Q.; Chen, J. Designing Quinone-Based Anodes with Rapid Kinetics for Rechargeable Proton Batteries. *Angewandte Chemie International Edition* **2022**, *61* (39). https://doi.org/10.1002/anie.202209642.

(15) Guo, Z.; Huang, J.; Dong, X.; Xia, Y.; Yan, L.; Wang, Z.; Wang, Y. An Organic/Inorganic Electrode-Based Hydronium-Ion Battery. *Nature Communications* **2020**, *11* (1), 959. https://doi.org/10.1038/s41467-020-14748-5.

(16) Bertaglia, T.; Kerr, E. F.; Sedenho, G. C.; Wong, A. A.; Colombo, R. N. P.; Macedo, L. J. A.; Iost, R. M.; Faria, L. C. I.; Lima, F. C. D. A.; Teobaldo, G. B. M.; Oliveira, C. L. P.; Aziz, M. J.; Gordon, R. G.; Crespilho, F. N. Self-Gelling Quinone-Based Wearable Microbattery. *Advanced Materials Technology* **2024**. https://doi.org/10.1002/admt.202400623.
(17) Pankratov, D.; Blum, Z.; Suyatin, D. B.; Popov, V. O.; Shleev, S. Self-Charging Electrochemical Biocapacitor. *ChemElectroChem* **2014**, *1* (2), 343–346. https://doi.org/10.1002/celc.201300142.
(18) Agnès, C.; Holzinger, M.; Le Goff, A.; Reuillard, B.; Elouarzaki, K.; Tingry, S.; Cosnier, S. Supercapacitor/Biofuel Cell Hybrids Based on Wired Enzymes on Carbon Nanotube Matrices: Autonomous Reloading after High Power Pulses in Neutral Buffered Glucose Solutions. *Energy Environmental Science* **2014**, *7* (6), 1884–1888. https://doi.org/10.1039/C3EE43986K.
(19) Pankratov, D.; Conzuelo, F.; Pinyou, P.; Alsaoub, S.; Schuhmann, W.; Shleev, S. A Nernstian Biosupercapacitor. *Angewandte Chemie International Edition* **2016**, *55* (49), 15434–15438. https://doi.org/10.1002/anie.201607144.
(20) Santoro, C.; Flores-Cadengo, C.; Soavi, F.; Kodali, M.; Merino-Jimenez, I.; Gajda, I.; Greenman, J.; Ieropoulos, I.; Atanassov, P. Ceramic Microbial Fuel Cells Stack: Power Generation in Standard and Supercapacitive Mode. *Scientific Reports* **2018**, *8* (1), 3281. https://doi.org/10.1038/s41598-018-21404-y.
(21) Kizling, M.; Draminska, S.; Stolarczyk, K.; Tammela, P.; Wang, Z.; Nyholm, L.; Bilewicz, R. Biosupercapacitors for Powering Oxygen Sensing Devices. *Bioelectrochemistry* **2015**, *106*, 34–40. https://doi.org/10.1016/j.bioelechem.2015.04.012.
(22) Narvaez Villarrubia, C. W.; Soavi, F.; Santoro, C.; Arbizzani, C.; Serov, A.; Rojas-Carbonell, S.; Gupta, G.; Atanassov, P. Self-Feeding Paper Based Biofuel Cell/Self-Powered Hybrid μ-Supercapacitor Integrated System. *Biosensors and Bioelectronics* **2016**, *86*, 459–465. https://doi.org/10.1016/j.bios.2016.06.084.
(23) Kizling, M.; Dzwonek, M.; Nowak, A.; Tymecki, Ł.; Stolarczyk, K.; Więckowska, A.; Bilewicz, R. Multi-Substrate Biofuel Cell Utilizing Glucose, Fructose and Sucrose as the Anode Fuels. *Nanomaterials* **2020**, *10* (8), 1534. https://doi.org/10.3390/nano10081534.
(24) González-Arribas, E.; Aleksejeva, O.; Bobrowski, T.; Toscano, M. D.; Gorton, L.; Schuhmann, W.; Shleev, S. Solar Biosupercapacitor. *Electrochemistry communications* **2017**, *74*, 9–13. https://doi.org/10.1016/j.elecom.2016.11.009.
(25) Park, T.; Lee, D. Y.; Ahn, B. J.; Kim, M.; Bok, J.; Kang, J.-S.; Lee, J. M.; Choi, C.; Jang, Y. Implantable Anti-Biofouling Biosupercapacitor with High Energy Performance. *Biosensors and Bioelectronics* **2024**, *243*, 115757. https://doi.org/10.1016/j.bios.2023.115757.
(26) Bollella, P.; Boeva, Z.; Latonen, R.-M.; Kano, K.; Gorton, L.; Bobacka, J. Highly Sensitive and Stable Fructose Self-Powered Biosensor Based on a Self-Charging Biosupercapacitor. *Biosensors and Bioelectronics* **2021**, *176*, 112909. https://doi.org/10.1016/j.bios.2020.112909.
(27) Sales, F. C. P. F.; Iost, R. M.; Martins, M. V. A.; Almeida, M. C.; Crespilho, F. N. An Intravenous Implantable Glucose/Dioxygen Biofuel Cell with Modified Flexible Carbon Fiber Electrodes. *Lab on a Chip* **2013**, *13* (3), 468–474. https://doi.org/10.1039/C2LC41007A.
(28) Rasmussen, M.; Ritzmann, R. E.; Lee, I.; Pollack, A. J.; Scherson, D. An Implantable Biofuel Cell for a Live Insect. *Journal of the American Chemical Society* **2012**, *134* (3), 1458–1460. https://doi.org/10.1021/ja210794c.
(29) Chen, T.; Barton, S. C.; Binyamin, G.; Gao, Z.; Zhang, Y.; Kim, H.-H.; Heller, A. A Miniature Biofuel Cell. *Journal of the American Chemical Society* **2001**, *123* (35), 8630–8631. https://doi.org/10.1021/ja0163164.

(30) Zebda, A.; Gondran, C.; Le Goff, A.; Holzinger, M.; Cinquin, P.; Cosnier, S. Mediatorless High-Power Glucose Biofuel Cells Based on Compressed Carbon Nanotube-Enzyme Electrodes. *Nature Communications* **2011**, *2* (1), 370. https://doi.org/10.1038/ncomms1365.
(31) Chaudhuri, S. K.; Lovley, D. R. Electricity Generation by Direct Oxidation of Glucose in Mediatorless Microbial Fuel Cells. *Nature Biotechnology* **2003**, *21* (10), 1229–1232. https://doi.org/10.1038/nbt867.
(32) Plumeré, N.; Rüdiger, O.; Oughli, A. A.; Williams, R.; Vivekananthan, J.; Pöller, S.; Schuhmann, W.; Lubitz, W. A Redox Hydrogel Protects Hydrogenase from High-Potential Deactivation and Oxygen Damage. *Nature Chemistry* **2014**, *6* (9), 822–827. https://doi.org/10.1038/nchem.2022.
(33) Gao, F.; Viry, L.; Maugey, M.; Poulin, P.; Mano, N. Engineering Hybrid Nanotube Wires for High-Power Biofuel Cells. *Nature Communications* **2010**, *1* (1), 2. https://doi.org/10.1038/ncomms1000.
(34) Kwon, C. H.; Lee, S.-H.; Choi, Y.-B.; Lee, J. A.; Kim, S. H.; Kim, H.-H.; Spinks, G. M.; Wallace, G. G.; Lima, M. D.; Kozlov, M. E.; Baughman, R. H.; Kim, S. J. High-Power Biofuel Cell Textiles from Woven Biscrolled Carbon Nanotube Yarns. *Nature Communications* **2014**, *5* (1), 3928. https://doi.org/10.1038/ncomms4928.
(35) Ruff, A.; Szczesny, J.; Marković, N.; Conzuelo, F.; Zacarias, S.; Pereira, I. A. C.; Lubitz, W.; Schuhmann, W. A Fully Protected Hydrogenase/Polymer-Based Bioanode for High-Performance Hydrogen/Glucose Biofuel Cells. *Nature Communications* **2018**, *9* (1), 3675. https://doi.org/10.1038/s41467-018-06106-3.
(36) Kwon, C. H.; Ko, Y.; Shin, D.; Kwon, M.; Park, J.; Bae, W. K.; Lee, S. W.; Cho, J. High-Power Hybrid Biofuel Cells Using Layer-by-Layer Assembled Glucose Oxidase-Coated Metallic Cotton Fibers. *Nature Communications* **2018**, *9* (1), 4479. https://doi.org/10.1038/s41467-018-06994-5.
(37) Wu, S.; Li, H.; Zhou, X.; Liang, P.; Zhang, X.; Jiang, Y.; Huang, X. A Novel Pilot-Scale Stacked Microbial Fuel Cell for Efficient Electricity Generation and Wastewater Treatment. *Water Research* **2016**, *98*, 396–403. https://doi.org/10.1016/j.watres.2016.04.043.
(38) Rossi, R.; Hur, A. Y.; Page, M. A.; Thomas, A. O.; Butkiewicz, J. J.; Jones, D. W.; Baek, G.; Saikaly, P. E.; Cropek, D. M.; Logan, B. E. Pilot Scale Microbial Fuel Cells Using Air Cathodes for Producing Electricity While Treating Wastewater. *Water Research* **2022**, *215*, 118208. https://doi.org/10.1016/j.watres.2022.118208.
(39) Pagnoncelli, K. C.; Pereira, A. R.; Sedenho, G. C.; Bertaglia, T.; Crespilho, F. N. Ethanol Generation, Oxidation and Energy Production in a Cooperative Bioelectrochemical System. *Bioelectrochemistry* **2018**, *122*, 11–25. https://doi.org/10.1016/j.bioelechem.2018.02.007.
(40) Blatter, M.; Delabays, L.; Furrer, C.; Huguenin, G.; Cachelin, C. P.; Fischer, F. Stretched 1000-L Microbial Fuel Cell. *Journal of Power Sources* **2021**, *483*, 229130. https://doi.org/10.1016/j.jpowsour.2020.229130.
(41) Hiegemann, H.; Herzer, D.; Nettmann, E.; Lübken, M.; Schulte, P.; Schmelz, K.-G.; Gredigk-Hoffmann, S.; Wichern, M. An Integrated 45 L Pilot Microbial Fuel Cell System at a Full-Scale Wastewater Treatment Plant. *Bioresource Technology* **2016**, *218*, 115–122. https://doi.org/10.1016/j.biortech.2016.06.052.
(42) Ren, H.; Tian, H.; Gardner, C. L.; Ren, T.-L.; Chae, J. A Miniaturized Microbial Fuel Cell with Three-Dimensional Graphene Macroporous Scaffold Anode Demonstrating a Record Power Density of over 10 000 W m^{-3}. *Nanoscale* **2016**, *8* (6), 3539–3547. https://doi.org/10.1039/C5NR07267K.
(43) Babanova, S.; Jones, J.; Phadke, S.; Lu, M.; Angulo, C.; Garcia, J.; Carpenter, K.; Cortese, R.; Chen, S.; Phan, T.; Bretschger, O. Continuous Flow, Large-scale, Microbial Fuel Cell System for the Sustained Treatment of Swine Waste. *Water Environment Research* **2020**, *92* (1), 60–72. https://doi.org/10.1002/wer.1183.

(44) Nath, D.; Ghangrekar, M. M. Plant Secondary Metabolites Induced Electron Flux in Microbial Fuel Cell: Investigation from Laboratory-to-Field Scale. *Scientific Reports* **2020**, *10* (1), 17185. https://doi.org/10.1038/s41598-020-74092-y.
(45) Park, Y.; Nguyen, V. K.; Park, S.; Yu, J.; Lee, T. Effects of Anode Spacing and Flow Rate on Energy Recovery of Flat-Panel Air-Cathode Microbial Fuel Cells Using Domestic Wastewater. *Bioresource Technology* **2018**, *258*, 57–63. https://doi.org/10.1016/j.biortech.2018.02.097.
(46) Ge, Z.; He, Z. Long-Term Performance of a 200 Liter Modularized Microbial Fuel Cell System Treating Municipal Wastewater: Treatment, Energy, and Cost. *Environmental Science: Water Research & Technology* **2016**, *2* (2), 274–281. https://doi.org/10.1039/C6EW00020G.
(47) Liang, P.; Duan, R.; Jiang, Y.; Zhang, X.; Qiu, Y.; Huang, X. One-Year Operation of 1000-L Modularized Microbial Fuel Cell for Municipal Wastewater Treatment. *Water Research* **2018**, *141*, 1–8. https://doi.org/10.1016/j.watres.2018.04.066.
(48) Dong, Y.; Qu, Y.; He, W.; Du, Y.; Liu, J.; Han, X.; Feng, Y. A 90-Liter Stackable Baffled Microbial Fuel Cell for Brewery Wastewater Treatment Based on Energy Self-Sufficient Mode. *Bioresource Technology* **2015**, *195*, 66–72. https://doi.org/10.1016/j.biortech.2015.06.026.
(49) Molenaar, S. D.; Mol, A. R.; Sleutels, T. H. J. A.; ter Heijne, A.; Buisman, C. J. N. Microbial Rechargeable Battery: Energy Storage and Recovery through Acetate. *Environmental Science & Technology Letters* **2016**, *3* (4), 144–149. https://doi.org/10.1021/acs.estlett.6b00051.
(50) Santos, M. S. S.; Peixoto, L.; Mushtaq, K.; Dias-Ferreira, C.; Mendes, A.; Alves, M. M. Bioelectrochemical Energy Storage in a Microbial Redox Flow Cell. *Jorunal of Energy Storage* **2021**, *39*, 102610. https://doi.org/10.1016/j.est.2021.102610.
(51) Li, S.; Fan, S.; Peng, X.; Zheng, D.; Li, D. Using Ferrous-Oxidizing Bacteria to Enhance the Performance of a PH Neutral All-Iron Flow Battery. *iScience* **2024**, *27* (1), 108595. https://doi.org/10.1016/j.isci.2023.108595.
